# Supplementary material for: Chemoselective synthesis and analysis of naturally occurring phosphorylated cysteine peptides
Source: Nat Commun. 2016 Sep 2;7:12703. doi: 10.1038/ncomms12703 (PMC5025809; doi:10.1038/ncomms12703)
Supplement: Supplementary Information — Supplementary Figures 1-61 and Supplementary Methods [file ncomms12703-s1.pdf]

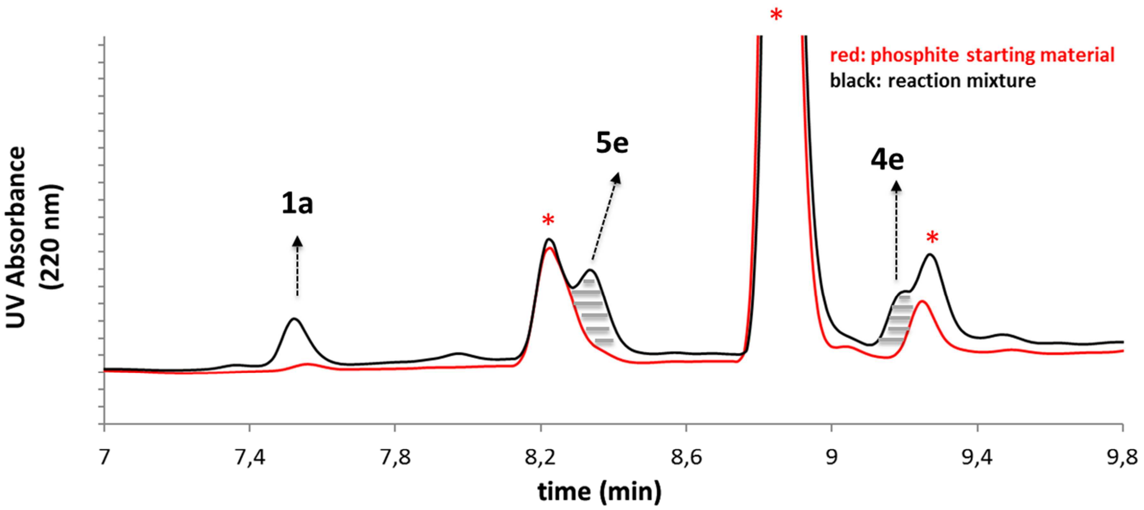

Supplementary Figure 1. Synthesis of phosphorothiolate esters peptides 4e and 5e upon reaction of peptide 2a with phosphite 3e in DMF (entry 5). Overlapping LC-UV chromatograms of reaction mixture and phosphite after 16h. Red asterisk mark the decomposition by-products of phosphite.

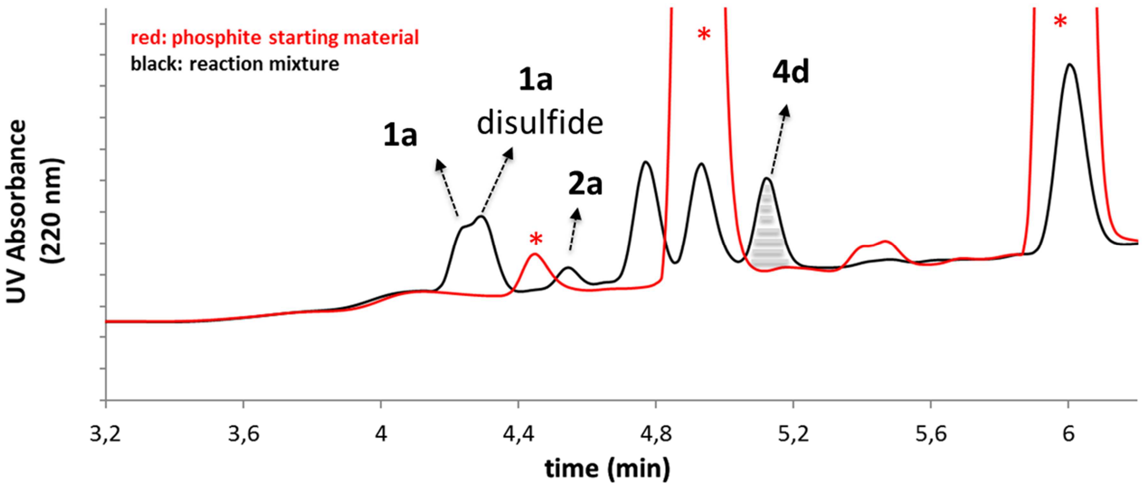

Supplementary Figure 2. Synthesis of phosphorothiolate ester peptide 4d upon reaction of peptide 2a with phosphite 3d in MeCN:Tris (3:2) (entry 9). Overlapping LC-UV chromatograms of reaction mixture and phosphite after 3h. Red asterisk mark the decomposition by-products of phosphite.

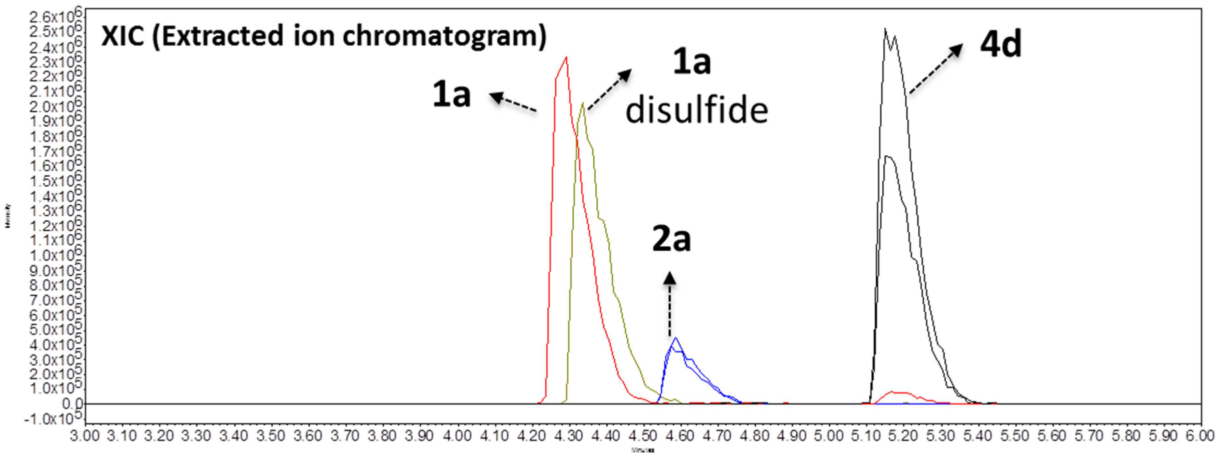

Supplementary Figure 3. Synthesis of phosphorothiolate ester peptide 4d upon reaction of peptide 2a with phosphite 3d in MeCN:Tris (3:2) (entry 9). XIC chromatogram with the extracted masses for peptide **1a**, **4d**, **2a** and disulfide **1a.**

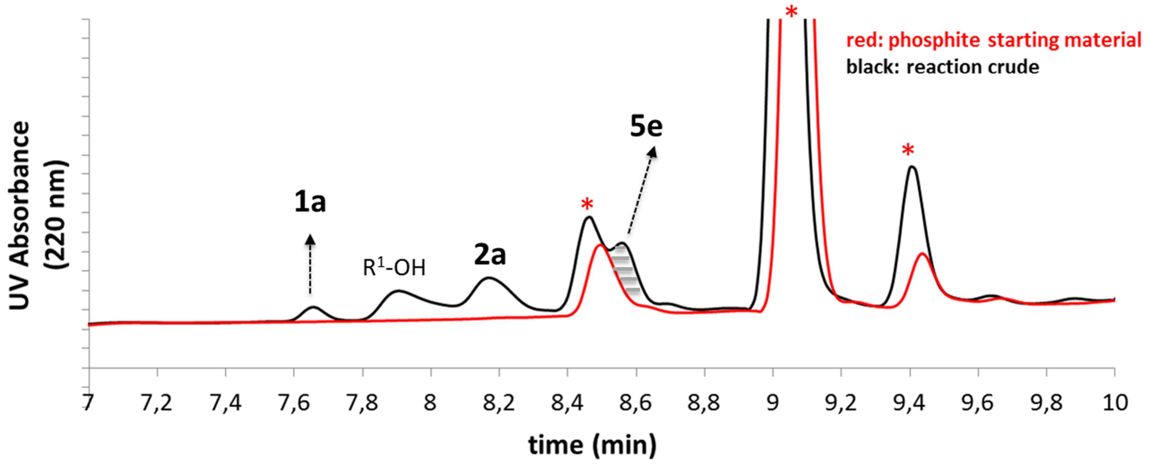

Supplementary Figure 4. Synthesis of phosphorothiolate ester peptide 5e upon reaction of peptide 2a with phosphite 3e in Tris-HCl buffer (entry 11). Overlapping LC-UV chromatograms of reaction mixture and phosphite after 3h. Red asterisk mark the decomposition by-products of phosphite.

a

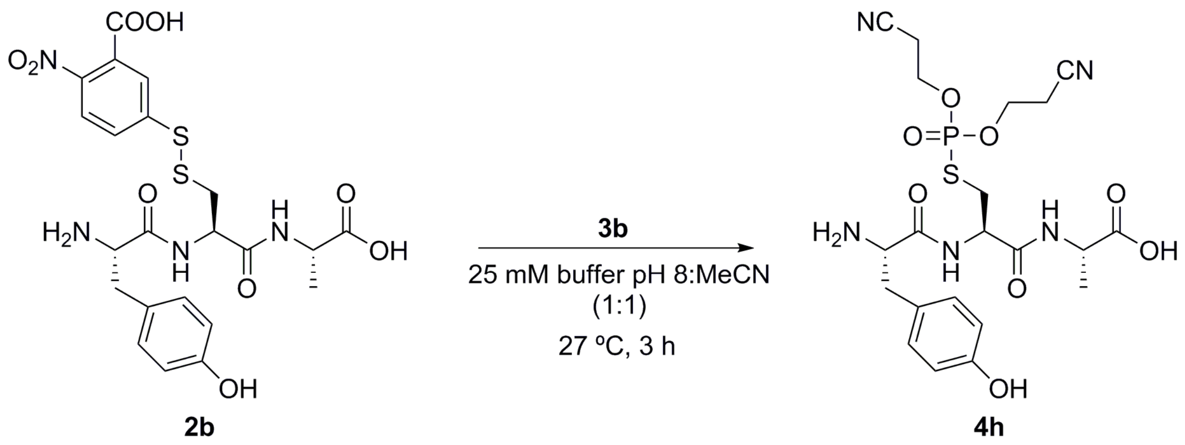

b

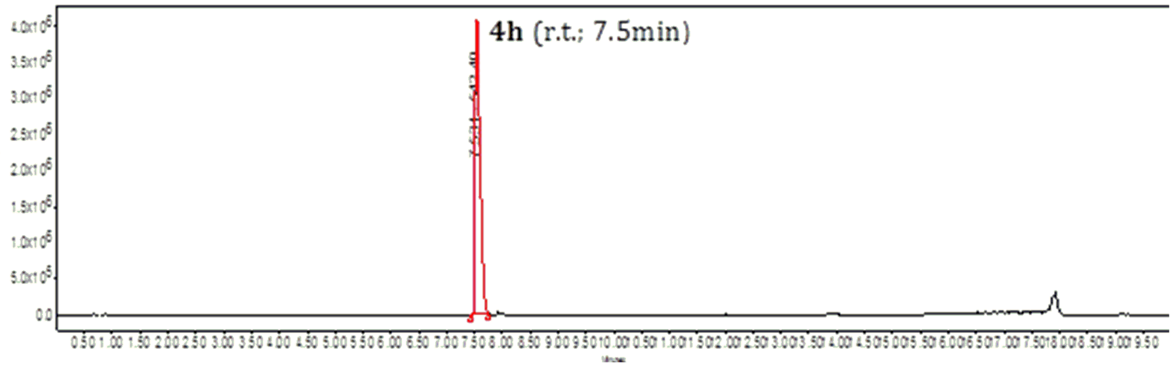

Supplementary Figure 5. Epimerization studies. (**a**) Synthesis of phosphorothiolate ester peptide **4h** from tripeptide **2b** and phosphite **3b**. (**b**) XIC (extracted ion chromatogram) with the m/z 542.1 for phosphorothiolate ester tripeptide **4h**. The XIC spectra show the presence of a single diastereoisomer (UPLC-retention time: 7.5 min).

a

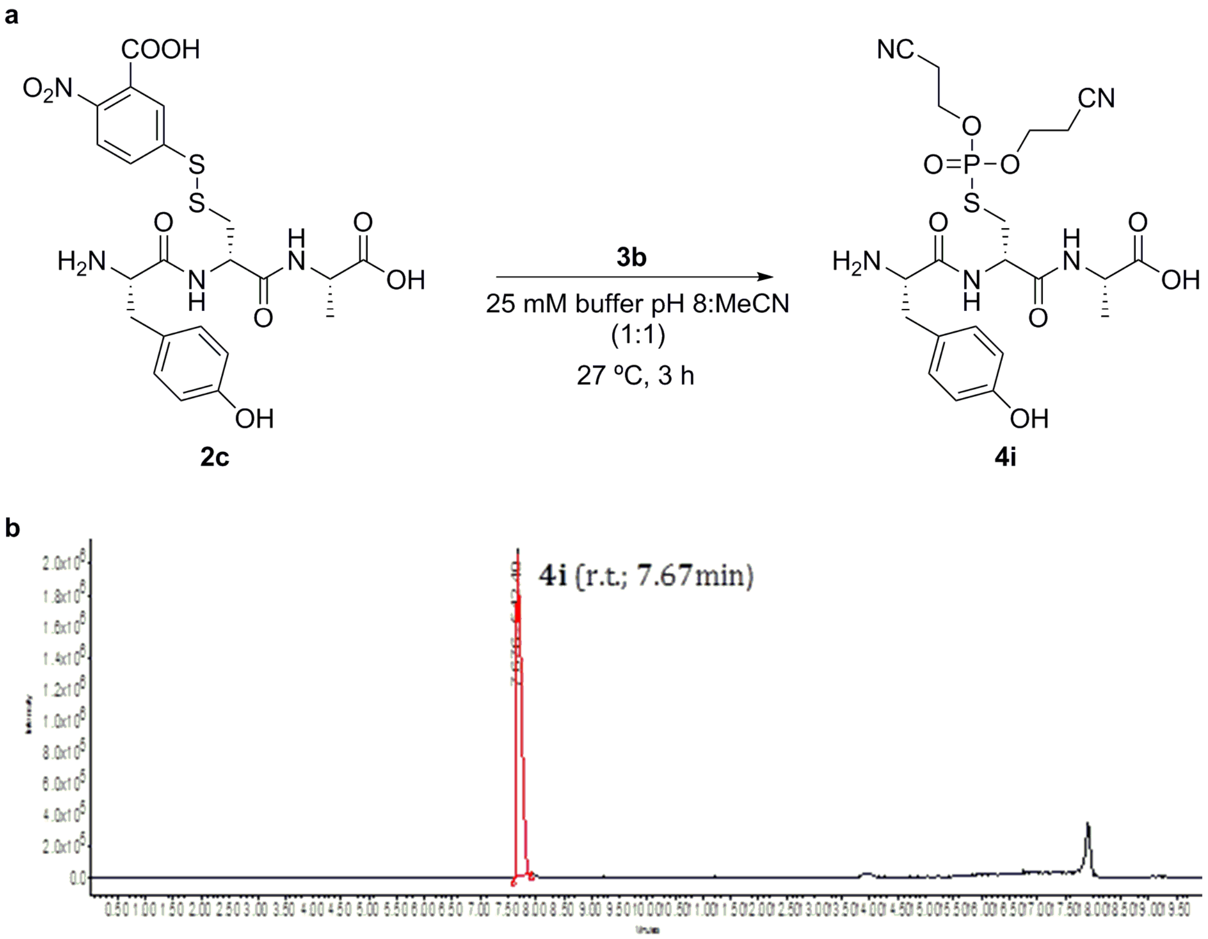

Supplementary Figure 6. Epimerization studies. (**a**) Synthesis of phosphorothiolate ester peptide **4i** from tripeptide **2c** and phosphite **3b**. (**b**) XIC (extracted ion chromatogram) with the m/z 542.1 for phosphorothiolate ester tripeptide **4i.** The XIC spectra show the presence of a single diastereoisomer (UPLC-retention time: 7.67 min).

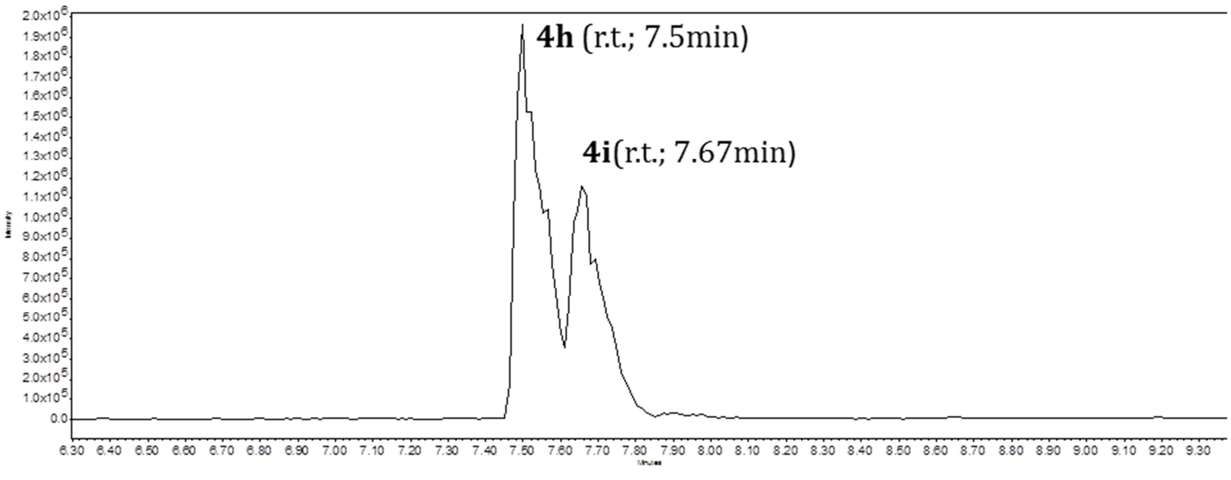

Supplementary Figure 7. Analysis of mixed reaction crudes (4h and 4i) by UPLC-UV and –MS. XIC (extracted ion chromatogram) with m/z 542.1. The mixture of both reaction crudes, i.e., **2b** with **3b** as well as **2c** with **3b**, shows two MS signals at 7.5 min and at 7.67 min after analysis by UPLC-UV and –MS which indicates the presence of two diastereoisomers.

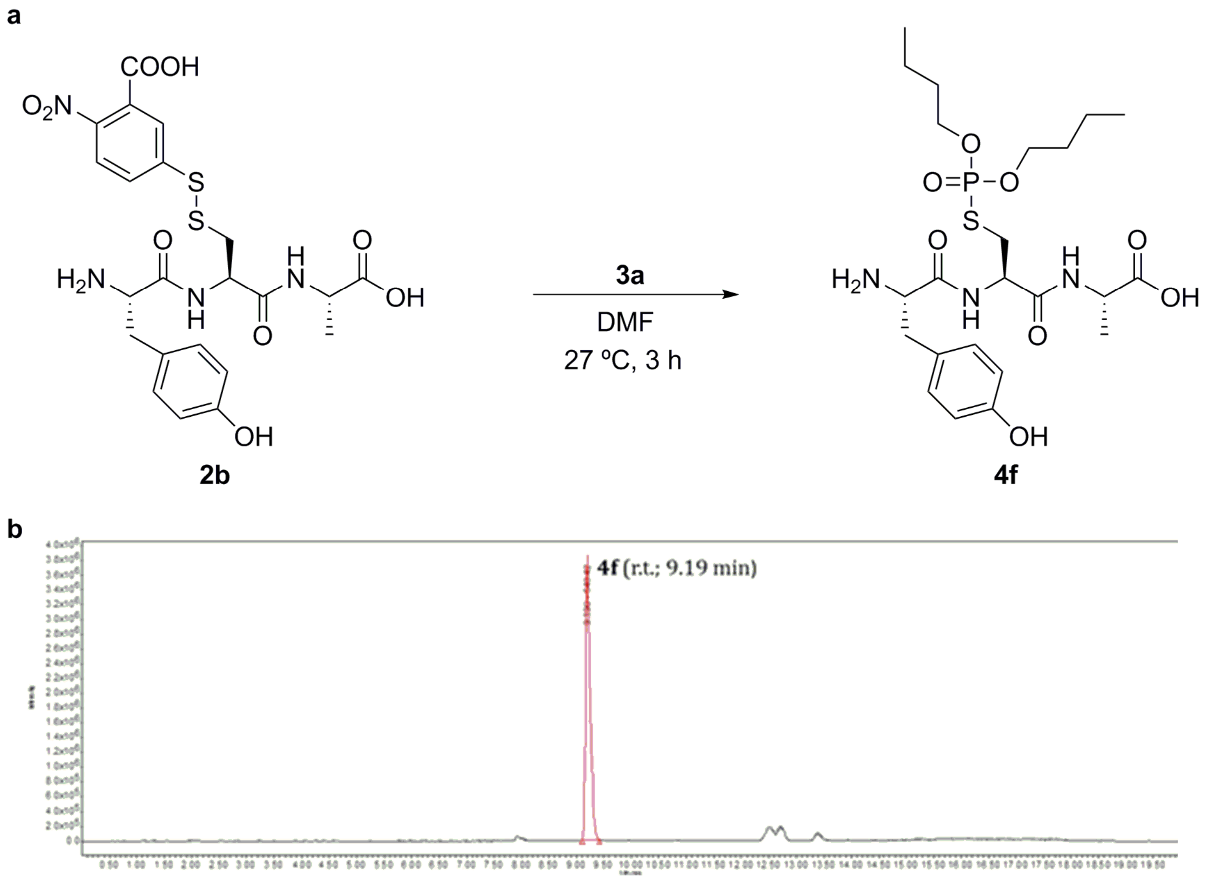

Supplementary Figure 8. Epimerization studies. (**a**) Synthesis of phosphorothiolate ester peptide **4f** from tripeptide **2b** and phosphite **3a**. (**b**) XIC (extracted ion chromatogram) with the m/z 548.3 for phosphorothiolate ester tripeptide **4f.** The XIC spectra show the presence of a single diastereoisomer (UPLC-retention time: 9.19 min).

a

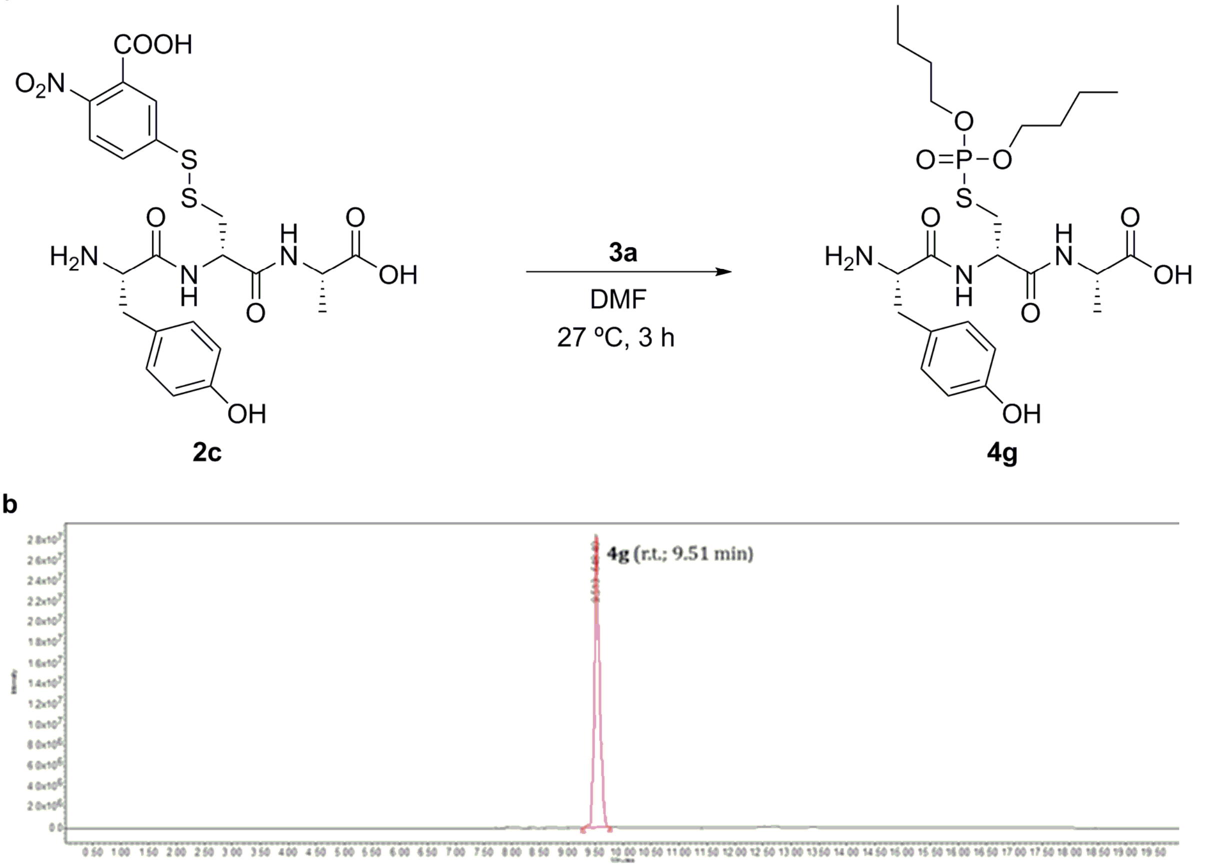

Supplementary Figure 9. Epimerization studies. (**a**) Synthesis of phosphorothiolate ester peptide **4g** from tripeptide **2c** and phosphite **3a**. (**b**) XIC (extracted ion chromatogram) with the m/z 548.3 for phosphorothiolate ester tripeptide **4g**. The XIC spectra show the presence of a single diastereoisomer (UPLC-retention time: 9.51 min).

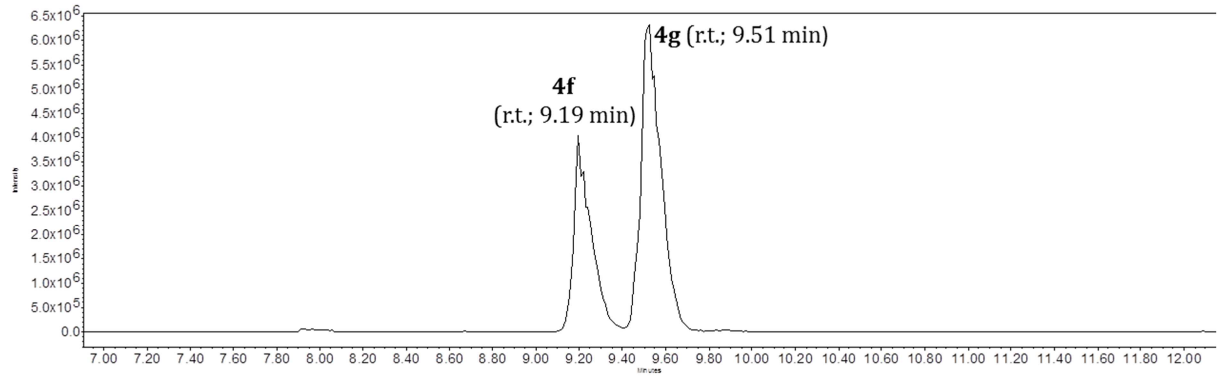

Supplementary Figure 10. Analysis of mixed reaction crudes (4f and 4g) by UPLC-UV and –MS. XIC (extracted ion chromatogram) with m/z 548.3. The mixture of both reaction crudes, i.e., **2b** with **3a** as well as **2c** with **3a**, shows two MS signals at 9.19 min and at 9.51 min after analysis by UPLC-UV and –MS which indicates the presence of two diastereoisomers.

a

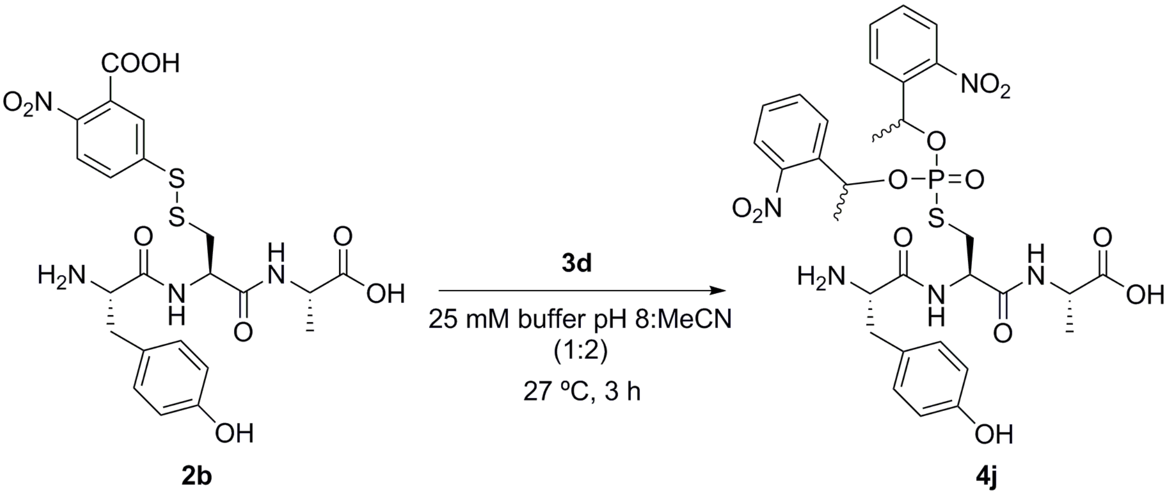

b

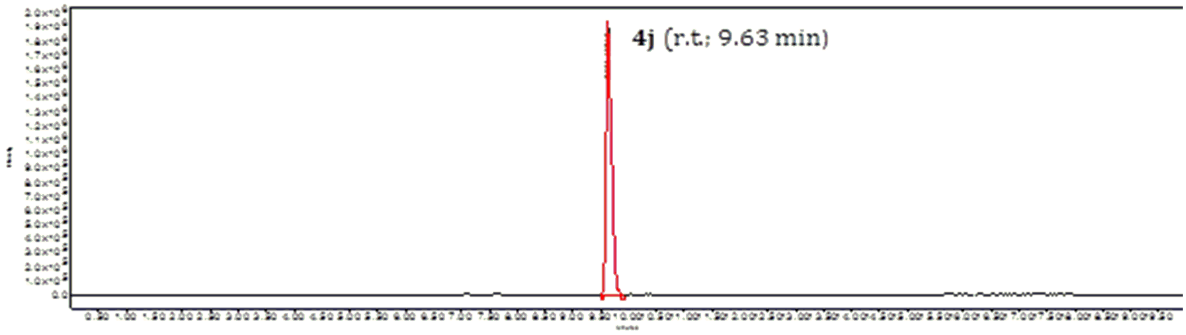

Supplementary Figure 11. Epimerization studies. (**a**) Synthesis of phosphorothiolate ester peptide **4j** from tripeptide **2b** and phosphite **3d**. (**b**) XIC (extracted ion chromatogram) with the m/z 734.2 for phosphorothiolate ester tripeptide **4j.** The XIC spectra show the presence of a single diastereoisomer (UPLC-retention time: 9.63 min).

a

b

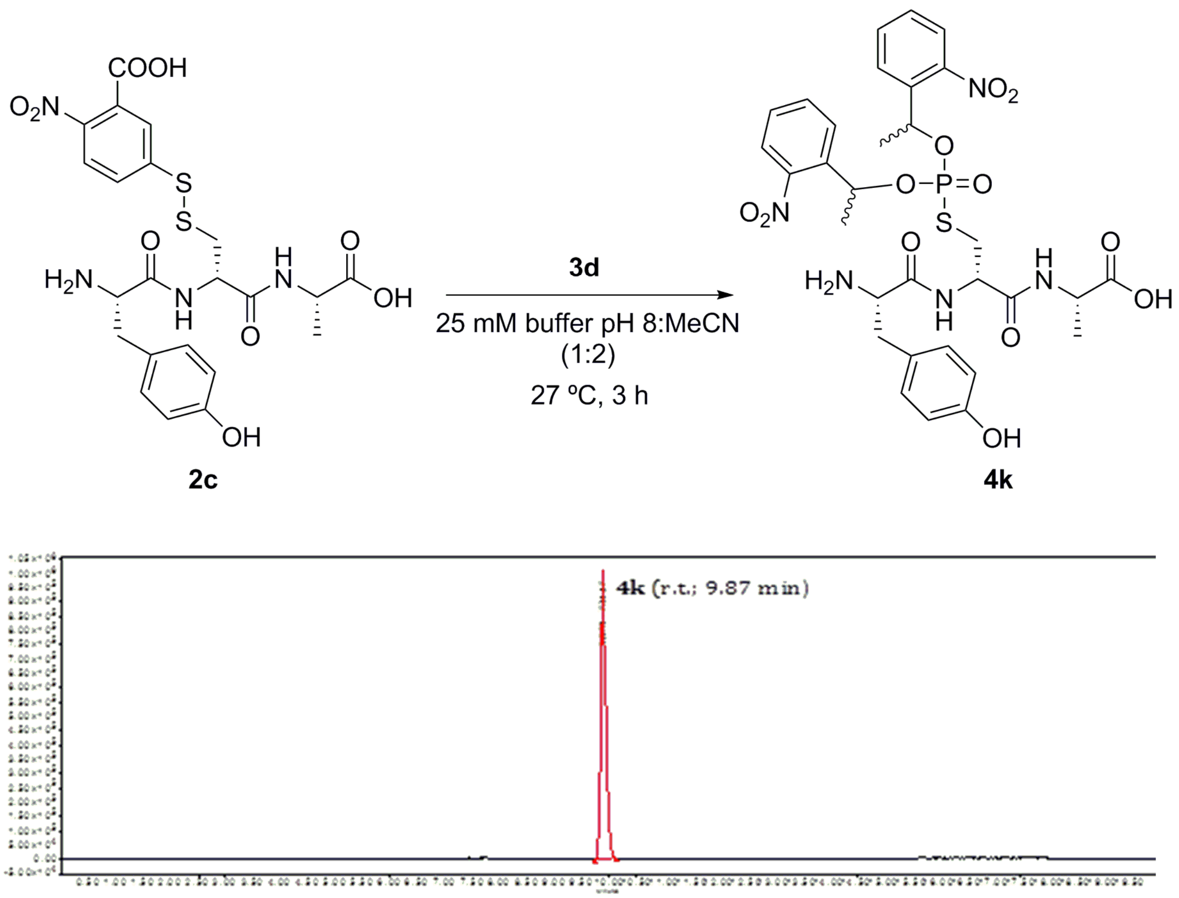

Supplementary Figure 12. Epimerization studies. (**a**) Synthesis of phosphorothiolate ester peptide **4k** from tripeptide **2c** and phosphite **3d**. (**b**) XIC (extracted ion chromatogram) with the m/z 734.2 for phosphorothiolate ester tripeptide **4k.** The XIC spectra show the presence of a single diastereoisomer (UPLC-retention time: 9.87 min).

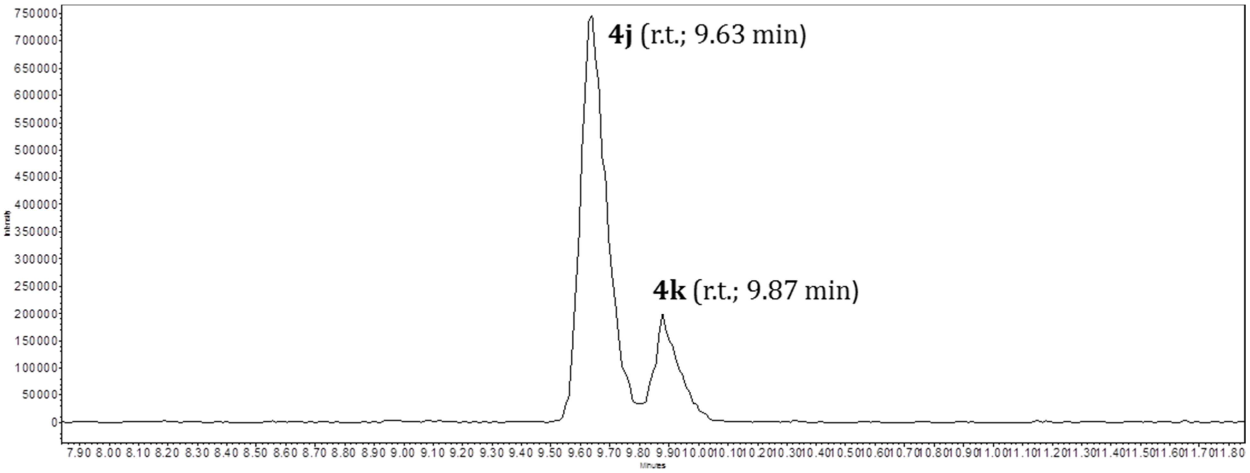

Supplementary Figure 13. Analysis of mixed reaction crudes (4j and 4k) by UPLC-UV and –MS. XIC (extracted ion chromatogram) with m/z 734.2. The mixture of both reaction crudes, i.e., **2b** with **3d** as well as **2c** with **3d**, shows two MS signals at 9.63 min and at 9.87 min after analysis by UPLC-UV and –MS which indicates the presence of two diastereoisomers.

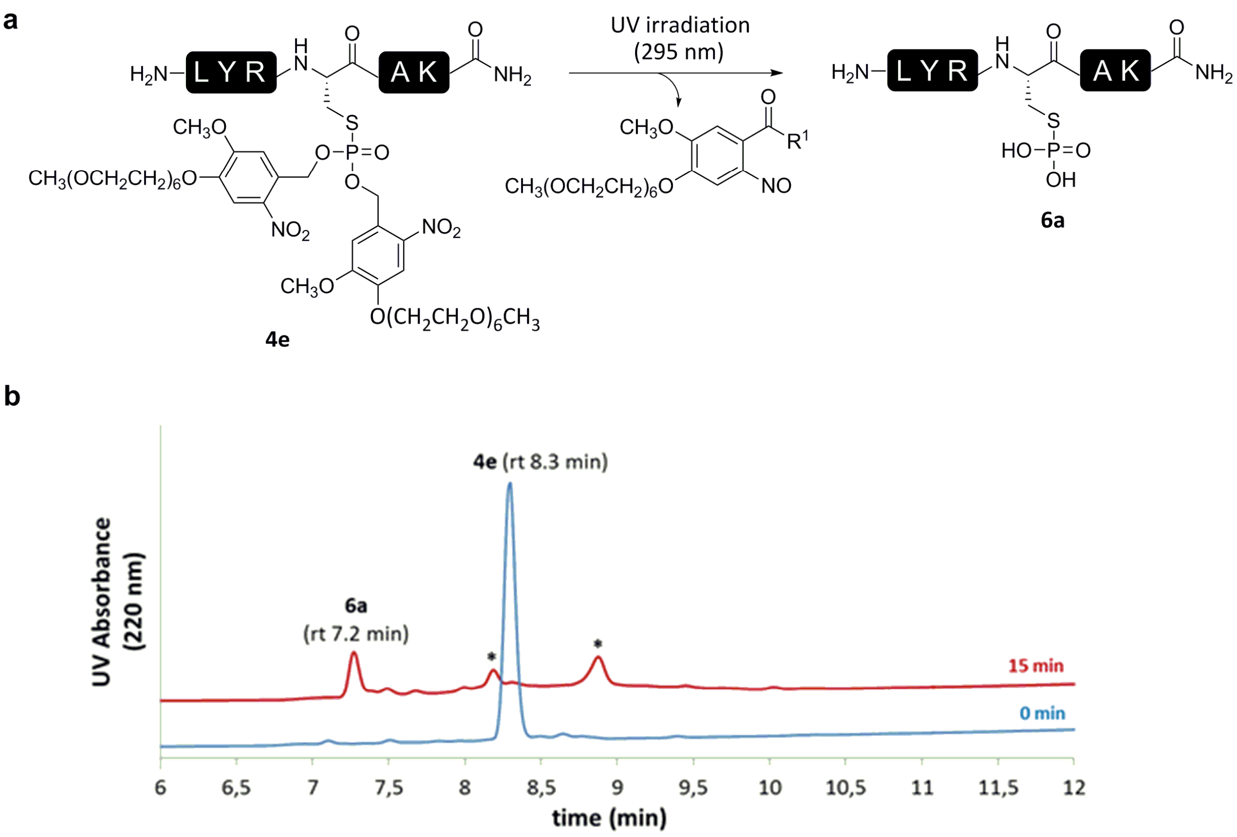

Supplementary Figure 14. UV-photodeprotection of phosphorothiolate ester peptide 4e. (**a**) Light-induced photolysis of peptide **4e** to furnish pCys peptide **6a**. (**b**) UPLC-UV trace before (blue) and after 15 min UV-irradiation at 295 nm (red) of **4e**.

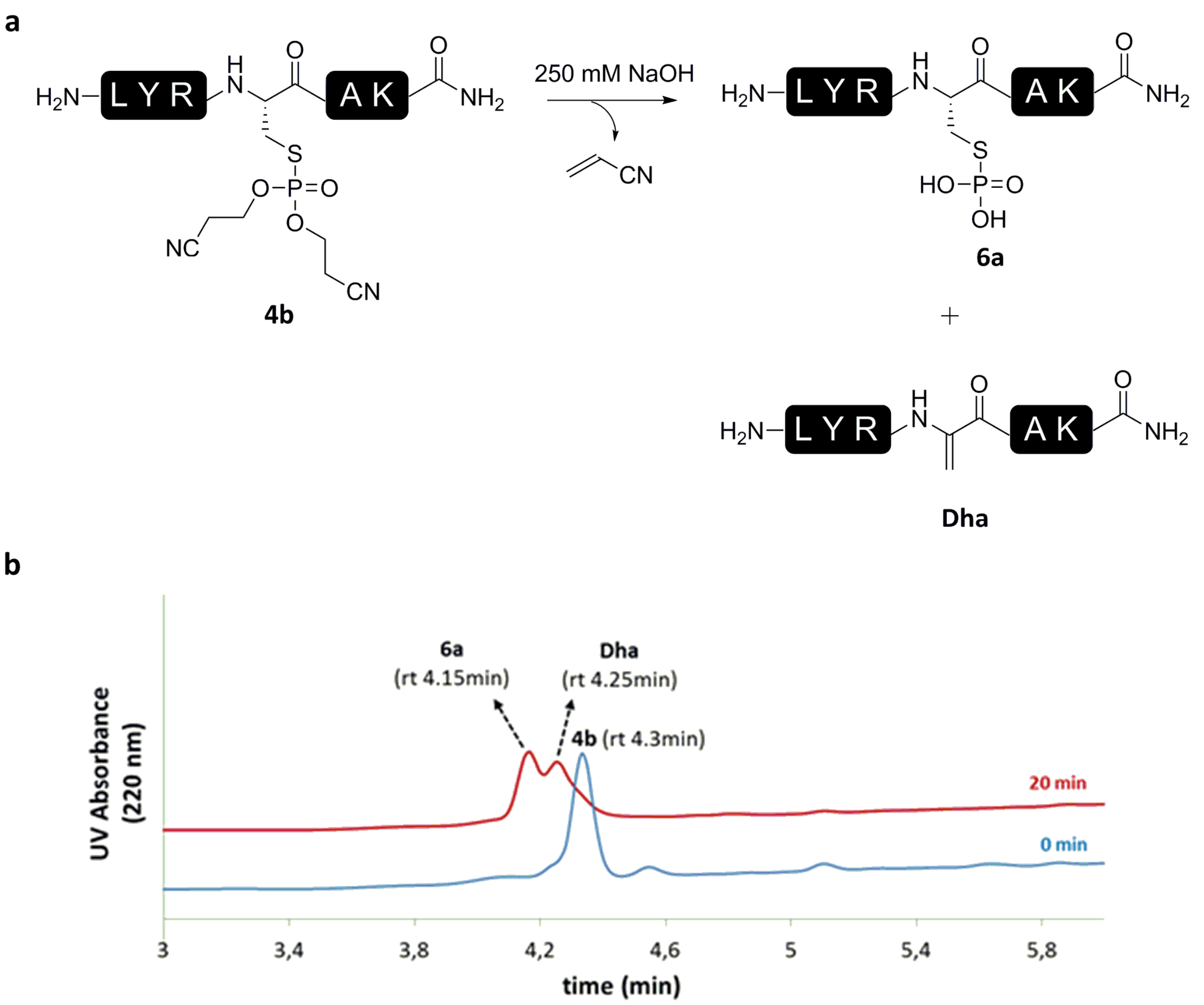

Supplementary Figure 15. Alkaline deprotection of peptide 4b. (**a**) An ammonium acetate solution (250 µL) of peptide **4b** (0.7 mg, 0.55 µmol) was incubated with 500 mM NaOH (250 µL) for 20 min. Final peptide concentration was 1.1 mM. β-elimination of bis(2-cyanoethyl) phosphorothiolate peptide **4b** furnish pCys peptide **6a** and dehydroalanine (**Dha**) peptide. (**b**) UPLC-UV before (blue) and after (red) 20 min incubation.

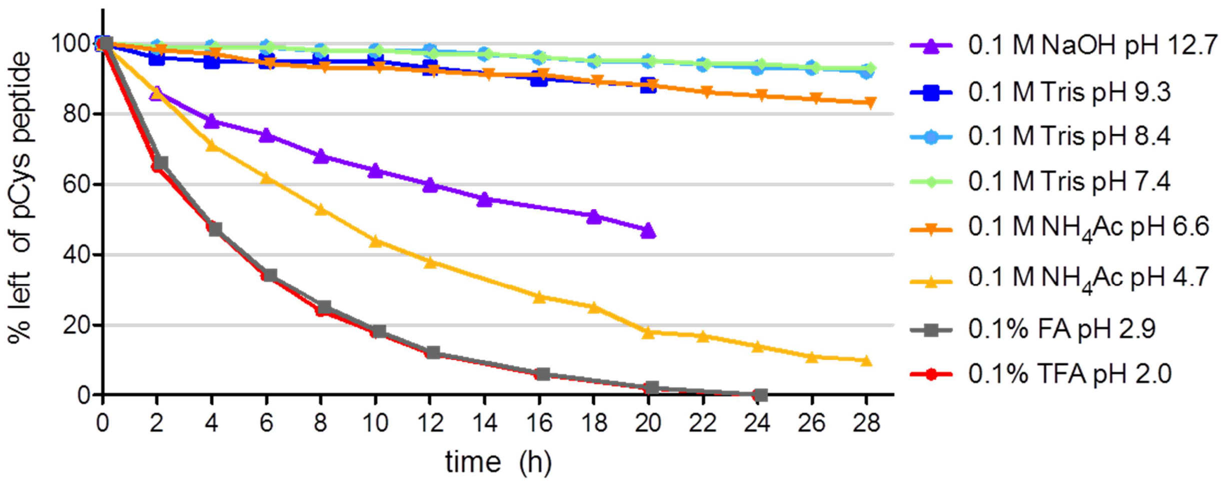

Supplementary Figure 16. Stability studies of pCys peptide 6a at different pHs. Peptide **6a** was incubated at acidic, neutral and basic pH and at room temperature and analysed by UPLC-UV (220 nm) at the time points indicated.

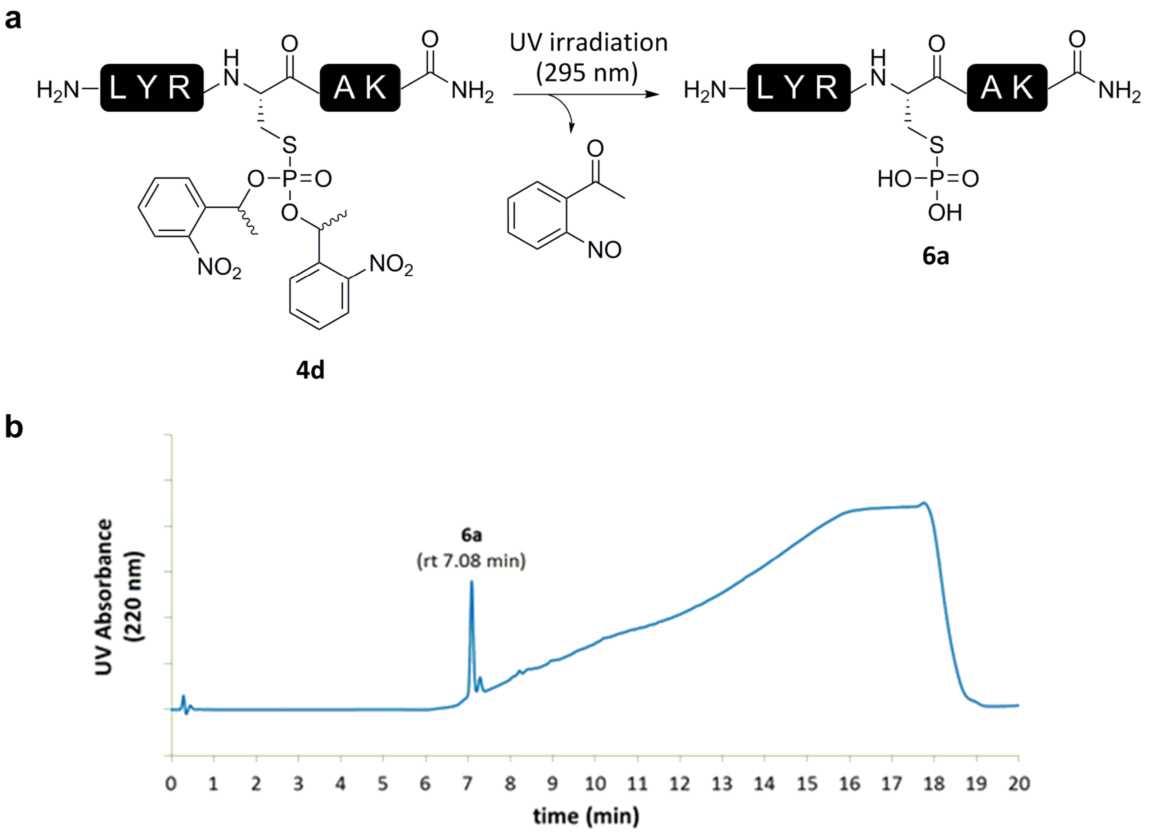

Supplementary Figure 17. UV-photodeprotection of phosphorothiolate ester peptide 4d. (**a**) Light-induced photolysis of peptide **4d** to furnish pCys peptide **6a**. (**b**) UPLC-UV trace of peptide **6a** after purification by semi-preparative HPLC using a MeCN/water gradient in alkaline aqueous buffer (pH 8.4) as a mobil phase. The side product detected by UPLC-UV and –MS at 7.2 min retention time, corresponds to the hydrolysis product of the phosphorothiolate cysteine-containing peptide, i.e., the unphosphorylated peptide. Integration of the unphosphorylated peptide by UV accounts for 9% relative to the phosphorothiolate cysteine-containing peptide.

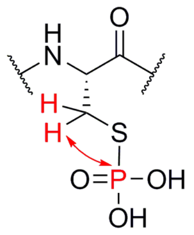

6a

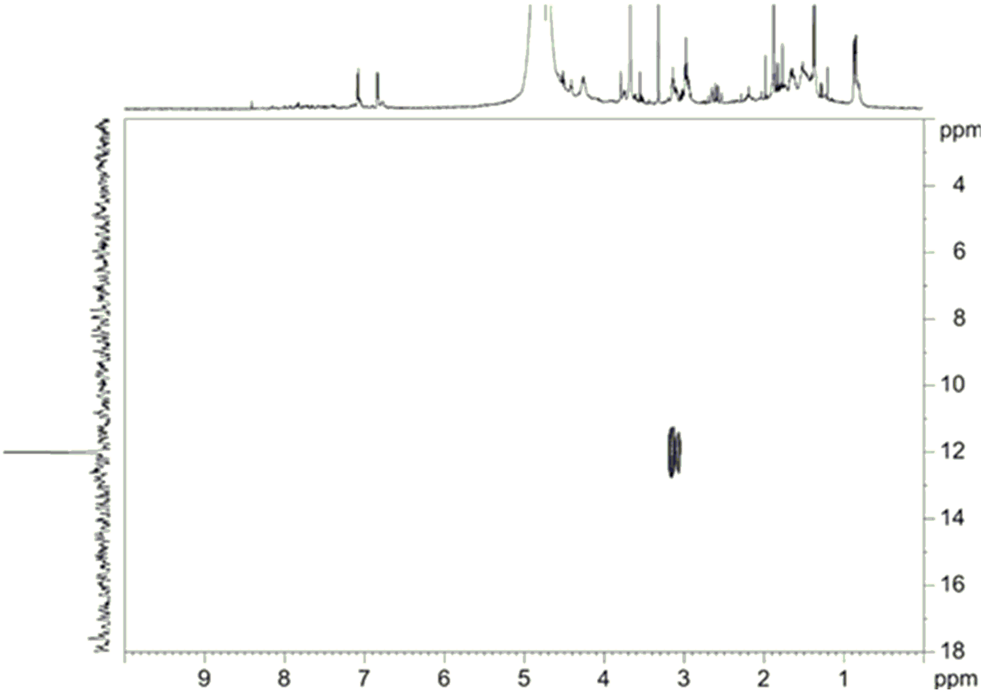

Supplementary Figure 18. ^{1}H, ^{31}P HMBC NMR experiment to characterize the phosphorylated residue in peptide 6a. The ^{31}P peak at 12.02 ppm showed coupling to the α-methylene hydrogen atoms in the pCys side chain at 3.13 ppm**.**

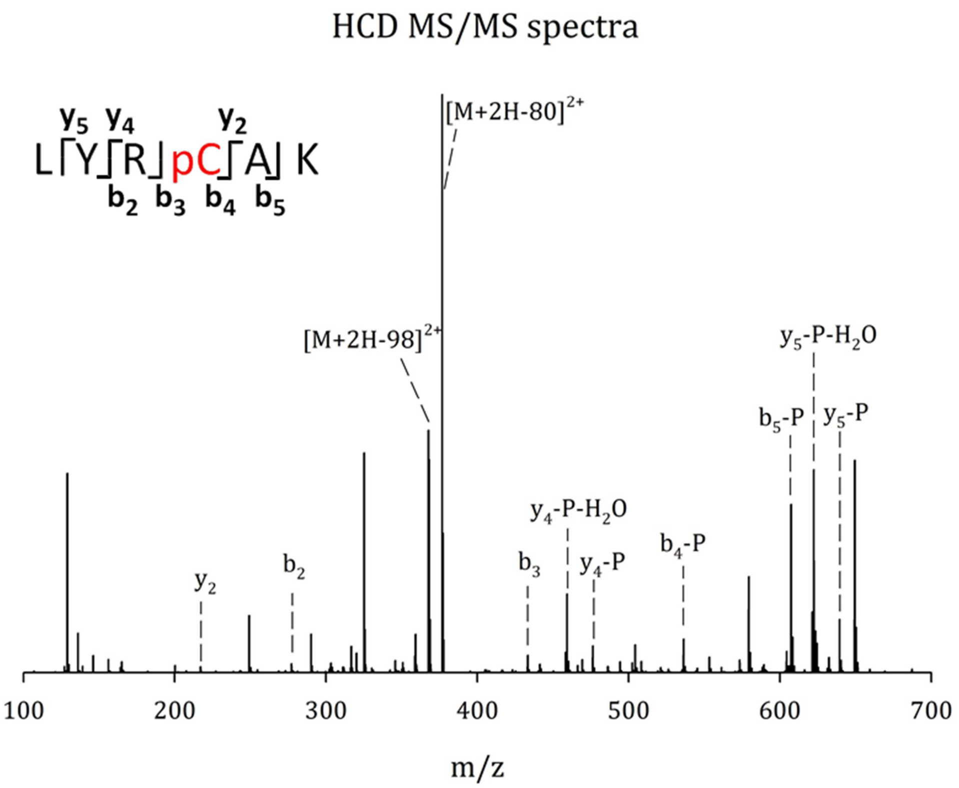

Supplementary Figure 19. Characterization by MS of peptide 6a. HCD MS/MS spectra of phosphorylated cysteine peptide **6a** showing complete neutral loss of phosphate and unphosphorylated *b*- and *y*-type fragments.

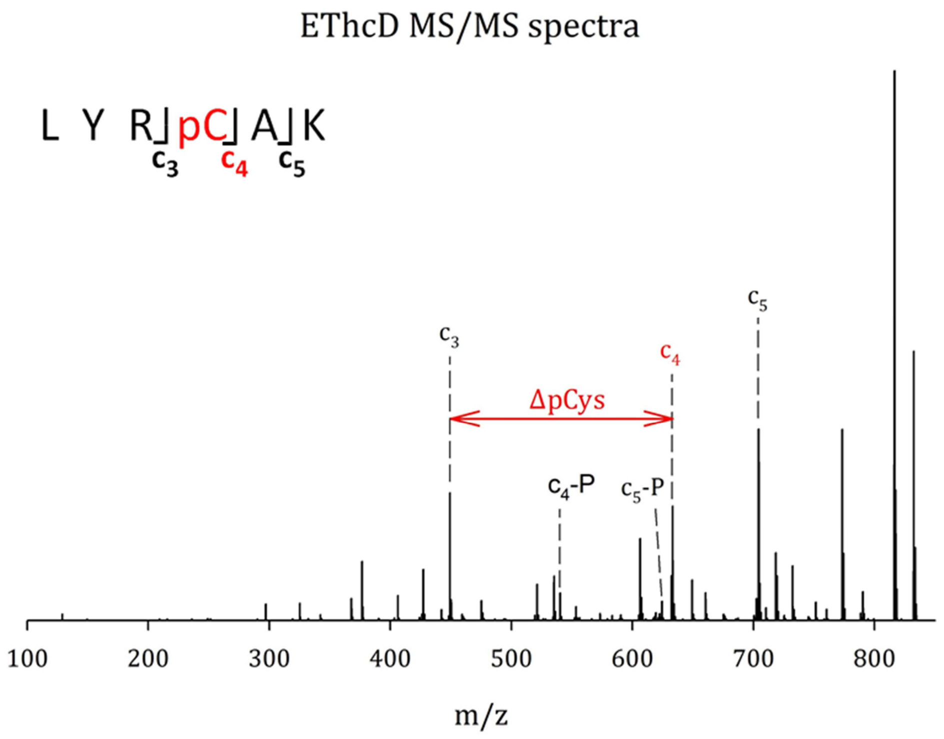

Supplementary Figure 20. Characterization by MS of peptide 6a. EThcD MS/MS spectra of phosphorylated cysteine peptide **6a** showing phosphorylated *c4* fragment ion.

a

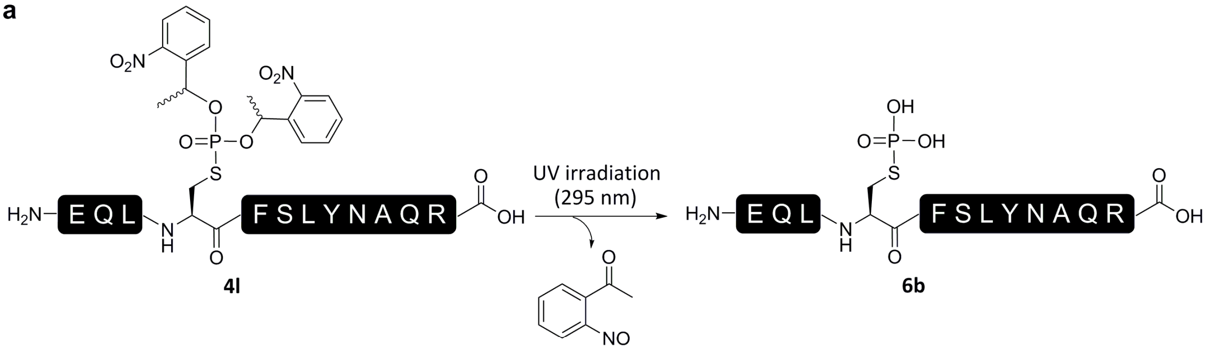

b

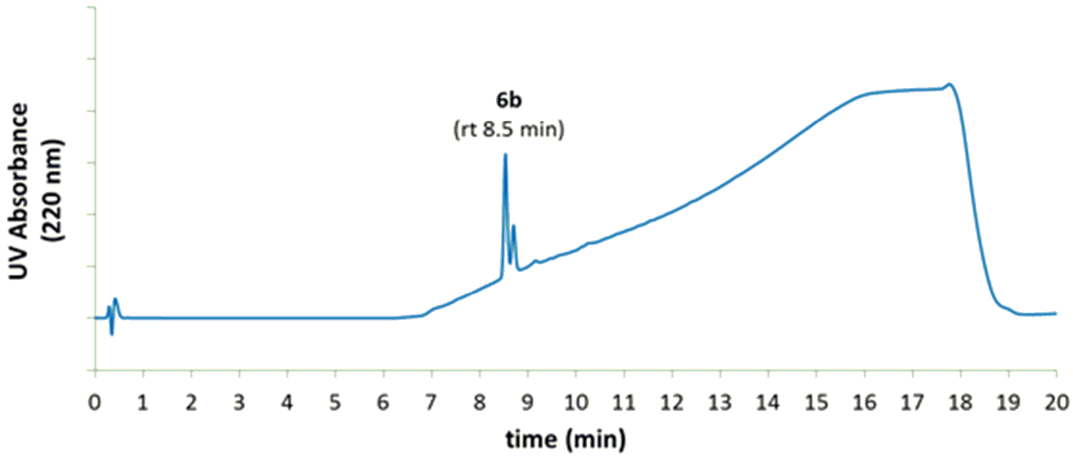

Supplementary Figure 21. UV irradiation of phosphorothiolate ester peptide 4l. (**a**) Light-induced photolysis of peptide **4l** to furnish pCys peptide **6b**. (**b**) UPLC-UV trace of peptide **6b** after purification by semi-preparative HPLC using a MeCN/water gradient in alkaline aqueous buffer (pH 8.4) as a mobil phase. The side product detected by UPLC-UV and –MS at 8.7 min retention time, corresponds to the hydrolysis product of the phosphorothiolate cysteine-containing peptide, i.e., the unphosphorylated peptide. Integration of the unphosphorylated peptide by UV accounts for 26% relative to the phosphorothiolate cysteine-containing peptide.

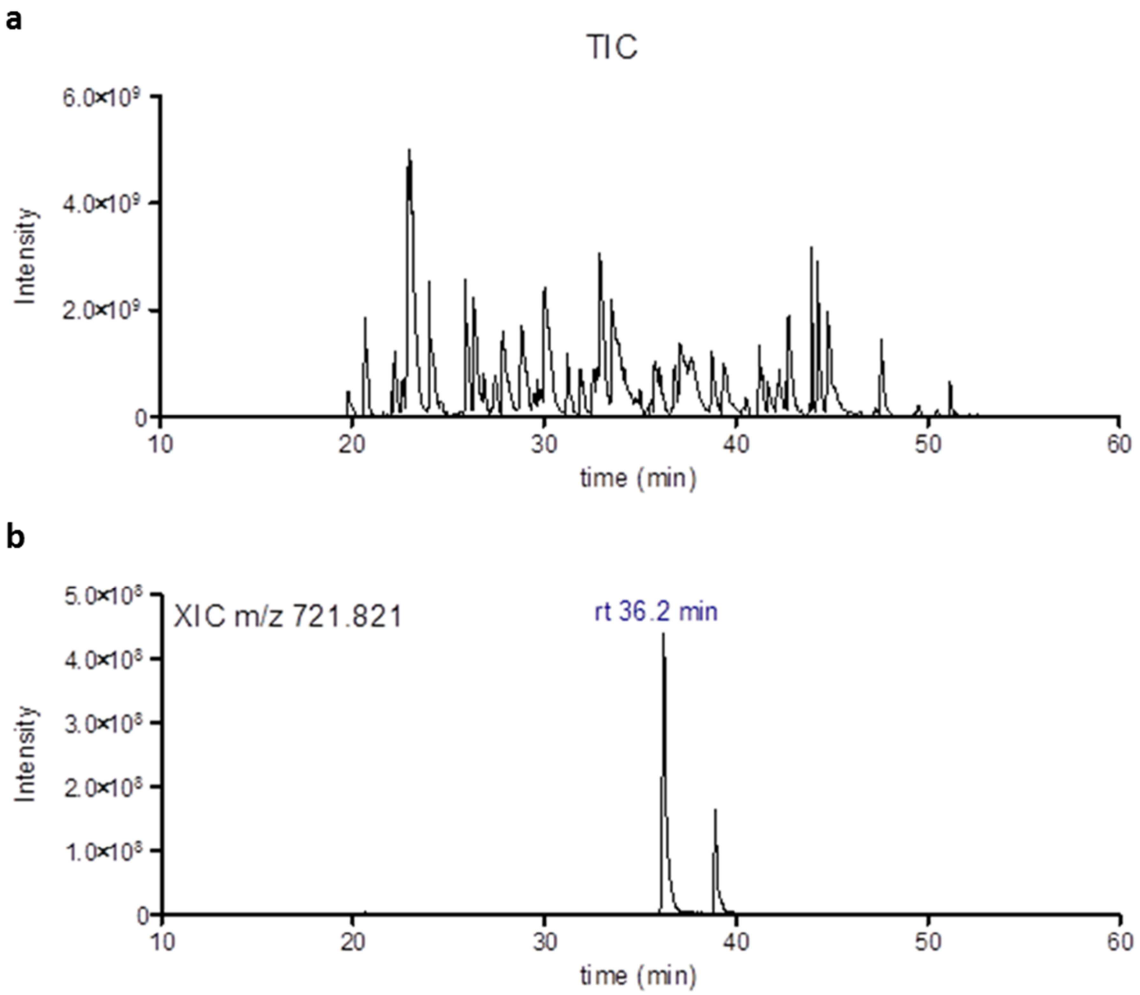

Supplementary Figure 22. Analysis by nLC-ESI-EThcD MS/MS of the endogenous pCys peptide ENITNLDApCITR by a bottom-up proteomic approach using SDS-PAGE protein separation in combination with in-gel tryptic digestion. (**a**) Total ion chromatogram of the digested gel band at 10 kDa. (**b**) Extracted ion chromatogram (XIC) of the pCys peptide of the digested gel band at 10 kDa.

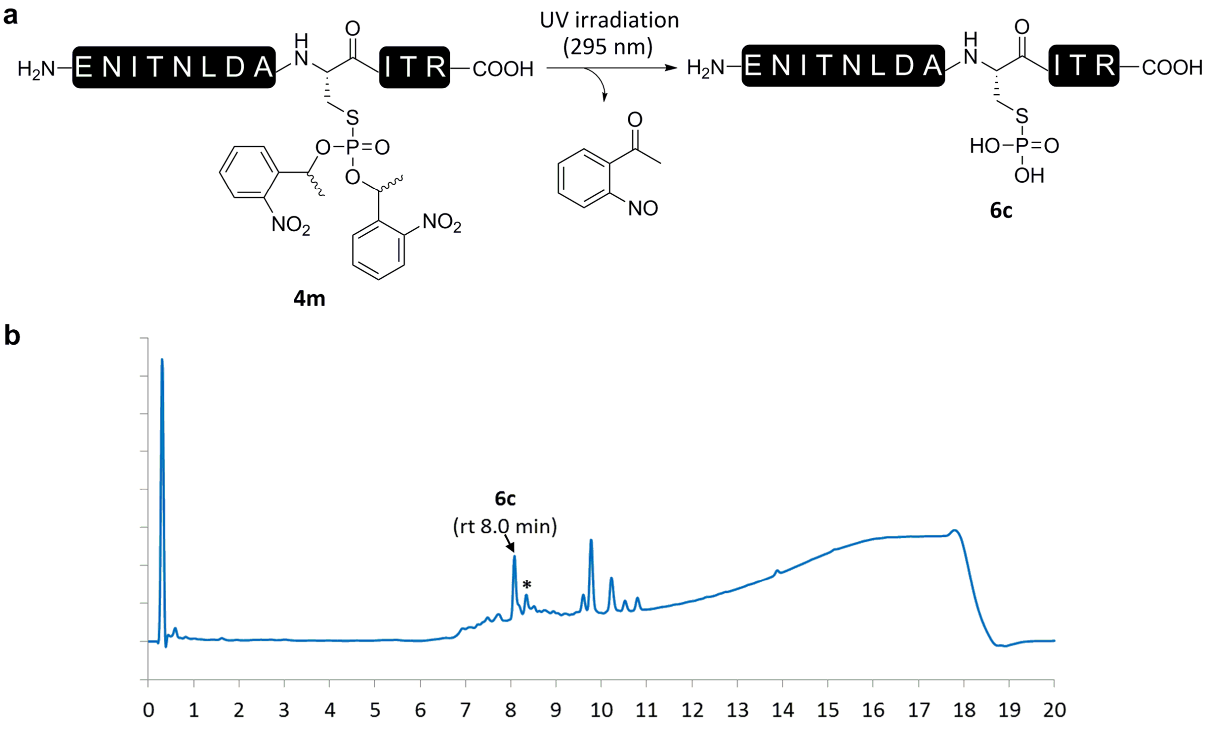

Supplementary Figure 23. UV-photodeprotection of phosphorothiolate ester peptide 4m. (**a**) Light-induced photolysis of peptide **4m** to furnish pCys peptide **6c**. (**b**) UPLC-UV trace directly after UV light exposure at 295 nm. *Side product detected by UPLC-UV and –MS at 8.3 min retention time, corresponds to the hydrolysis product of the phosphorothiolate cysteine-containing peptide, i.e., the unphosphorylated peptide. The poor UV-intensity is due to the absence of UV-active amino acids on the peptide sequence.

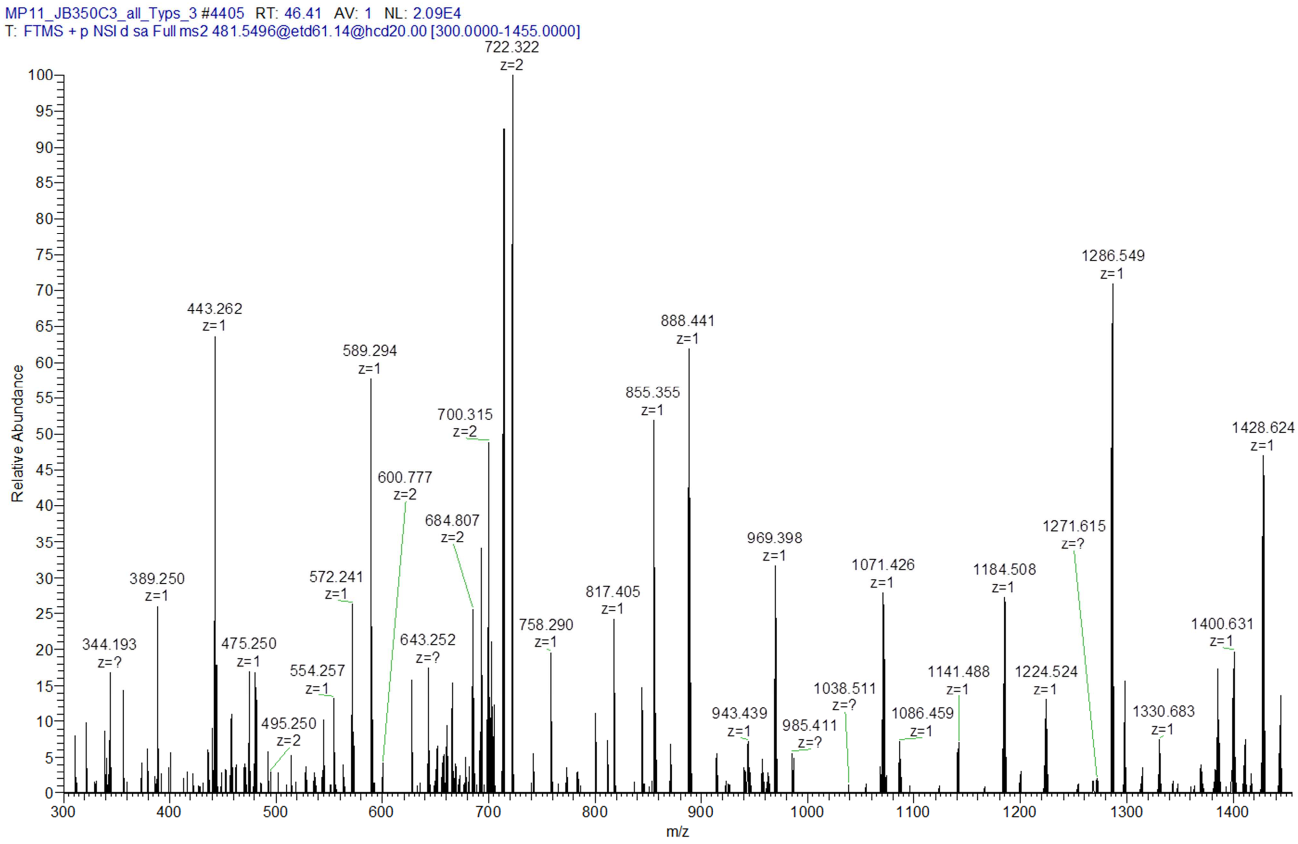

Supplementary Figure 24. Characterization by MS of synthetic peptide 6c. EThcD MS/MS spectra of phosphorylated cysteine peptide **6c** showing complete sequence coverage and diagnostic fragment ions c9, y4 and z5.

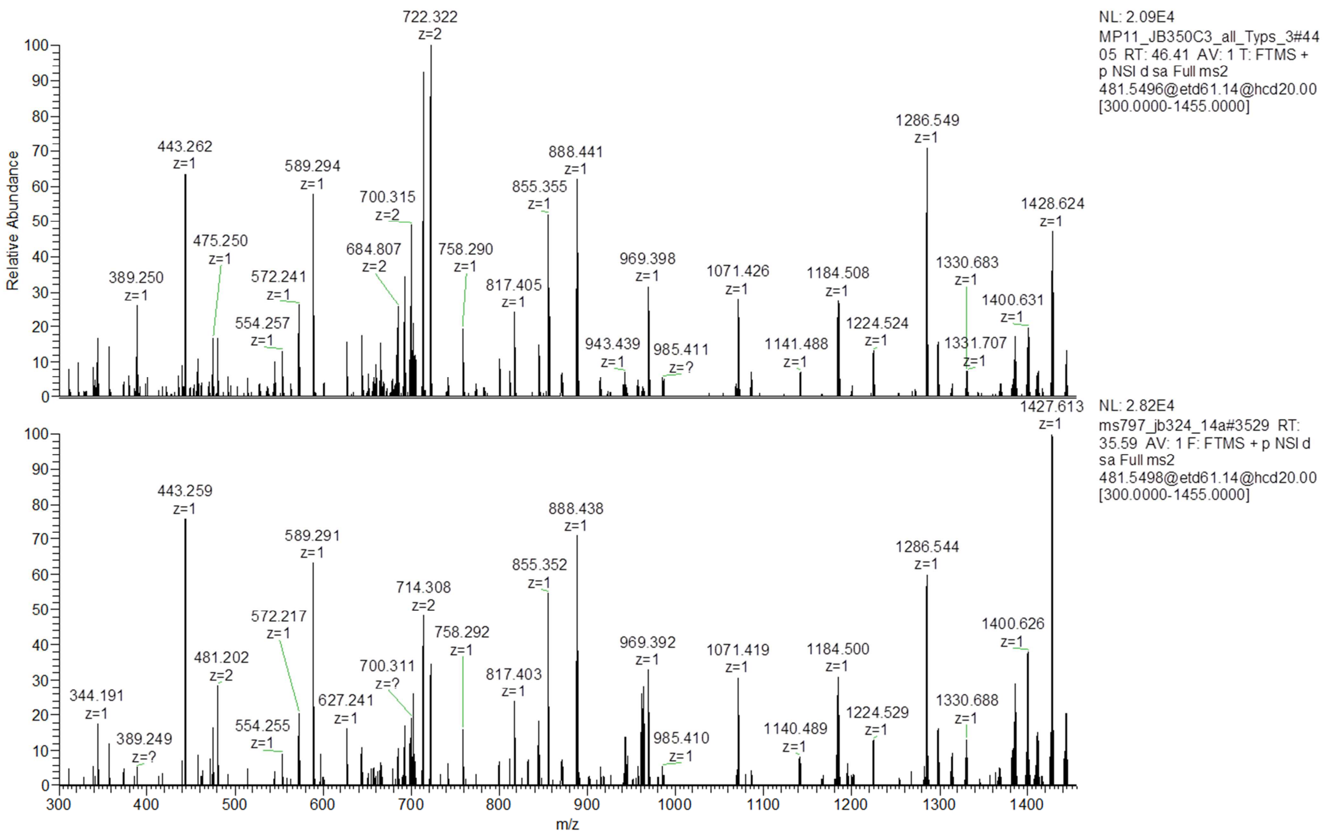

Supplementary Figure 25. Comparision of EThcD MS/MS spectra of synthetic peptide 6c (top) and endogenous pCys peptide (bottom) ENITNLDApCITR.

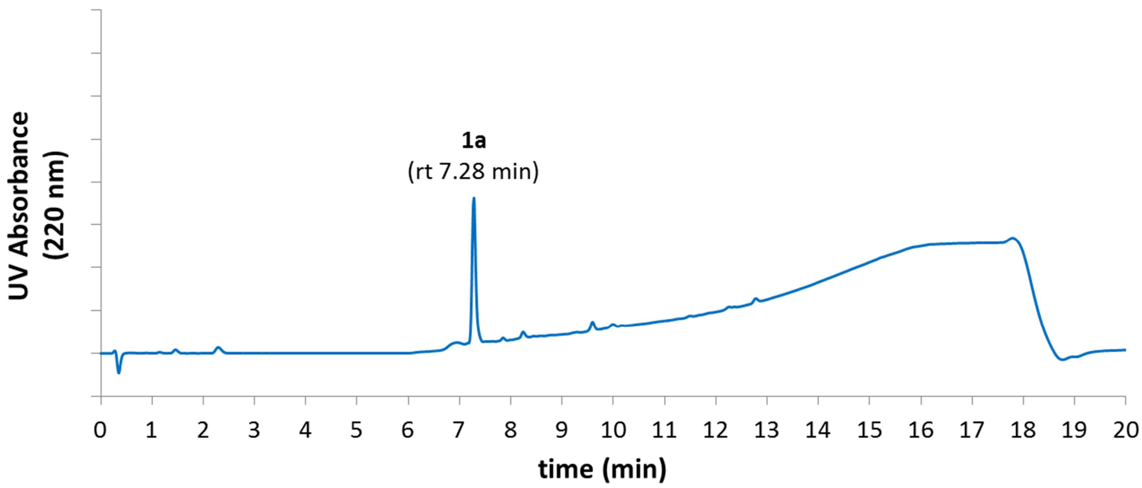

Supplementary Figure 26. UPLC-UV trace of pure peptide **1a** after purification by semi-preparative HPLC using a TFA gradient.

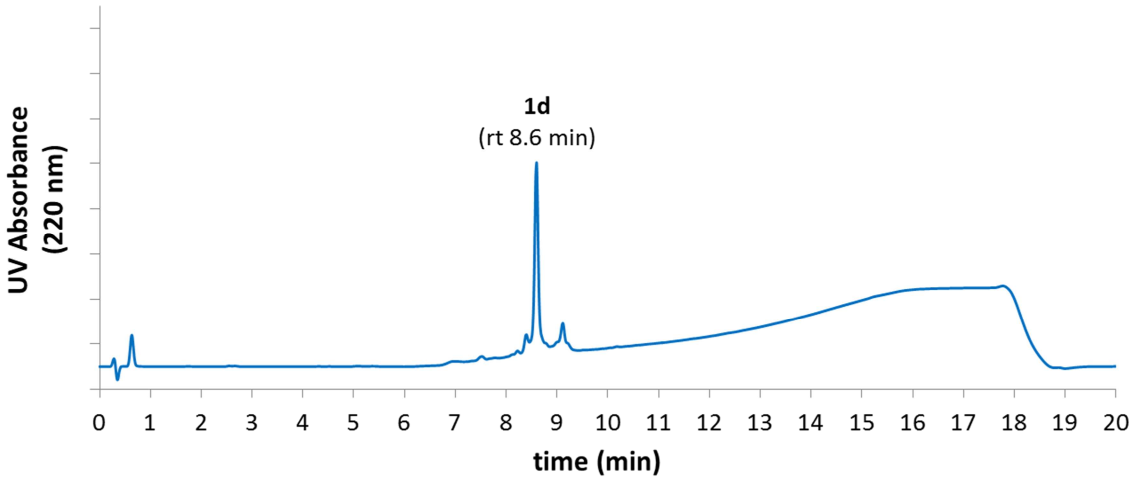

Supplementary Figure 27. UPLC-UV trace of pure peptide **1d** after purification by semi-preparative HPLC using a TFA gradient.

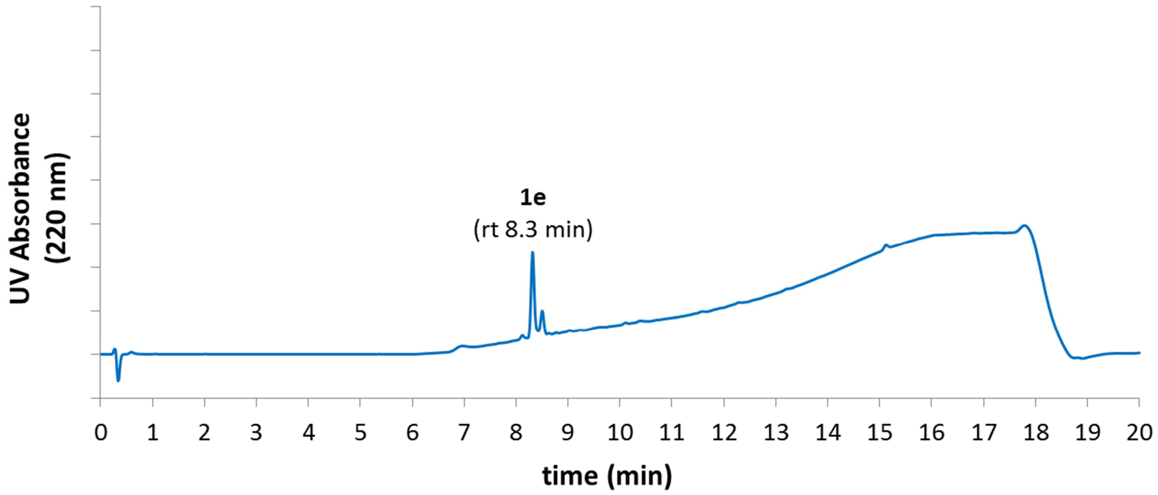

Supplementary Figure 28. UPLC-UV trace of peptide **1e** after purification by semi-preparative HPLC using a TFA gradient.

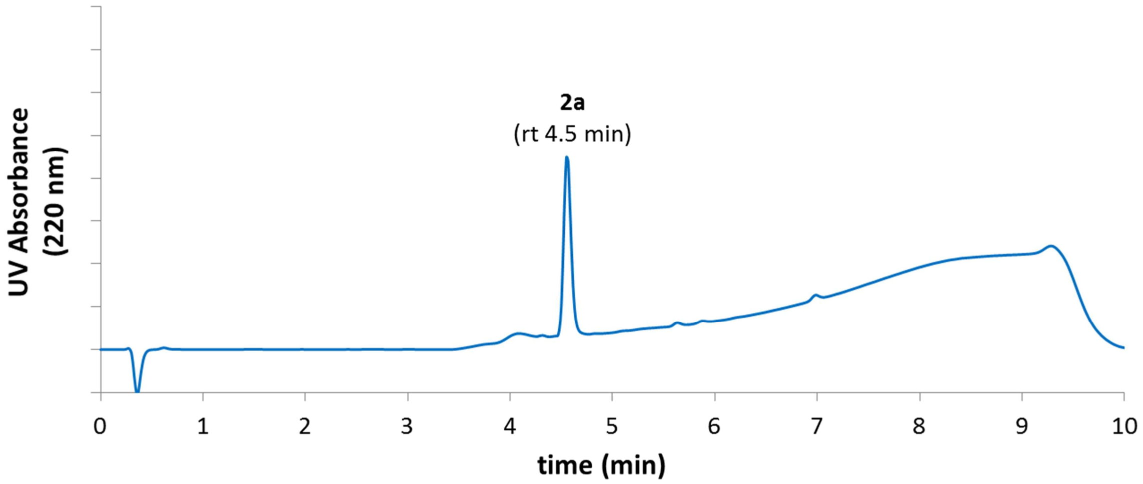

Supplementary Figure 29. UPLC-UV trace of pure peptide **2a** after purification by semi-preparative HPLC using a TFA gradient.

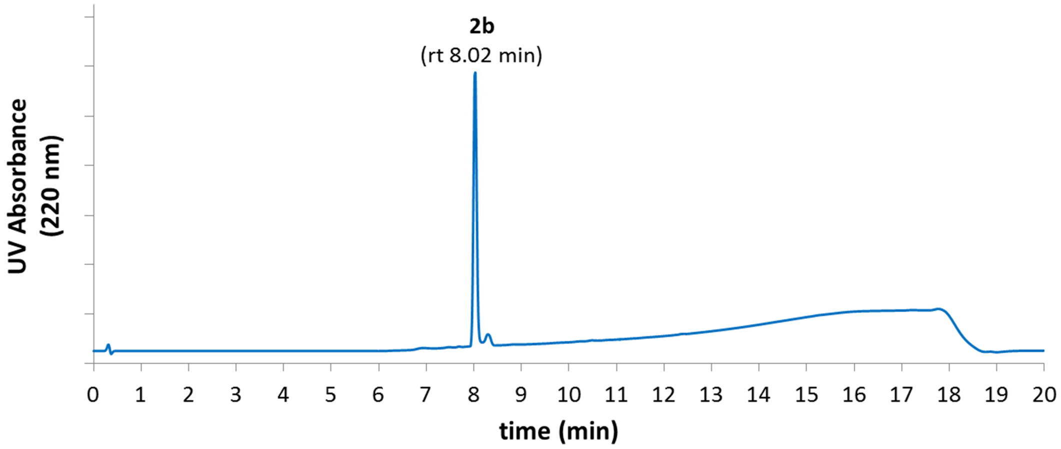

Supplementary Figure 30. UPLC-UV trace of pure peptide **2b** after purification by semi-preparative HPLC using a TFA gradient.

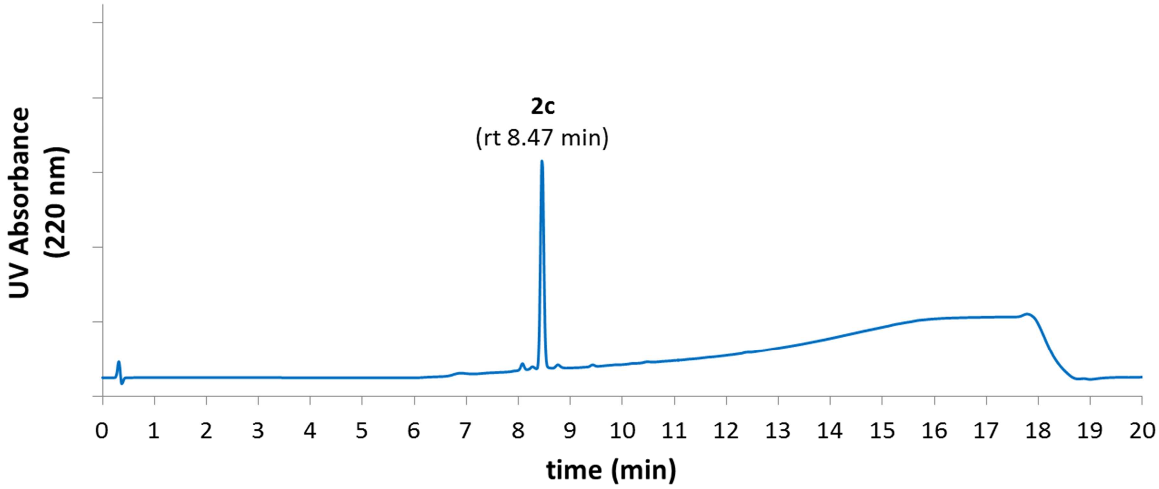

Supplementary Figure 31. UPLC-UV trace of pure peptide **2c** after purification by semi-preparative HPLC using a TFA gradient.

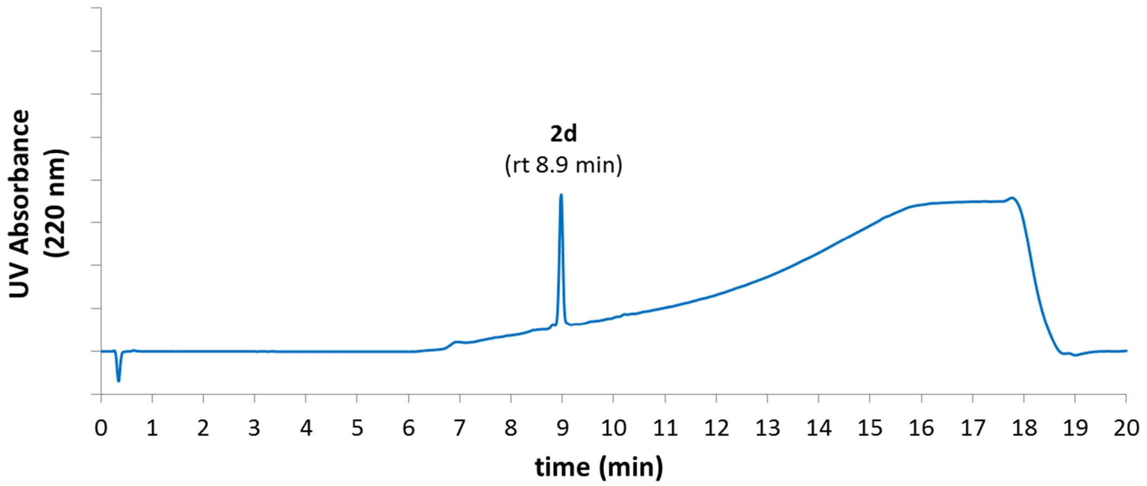

Supplementary Figure 32. UPLC-UV trace of pure peptide **2d** after purification by semi-preparative HPLC using a TFA gradient.

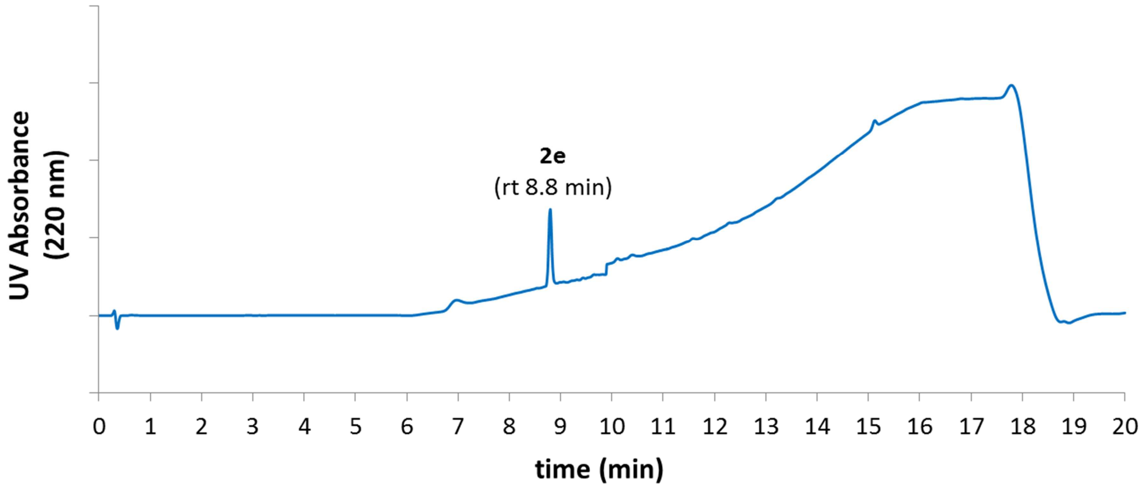

Supplementary Figure 33. UPLC-UV trace of pure peptide **2e** after purification by semi-preparative HPLC using a TFA gradient.

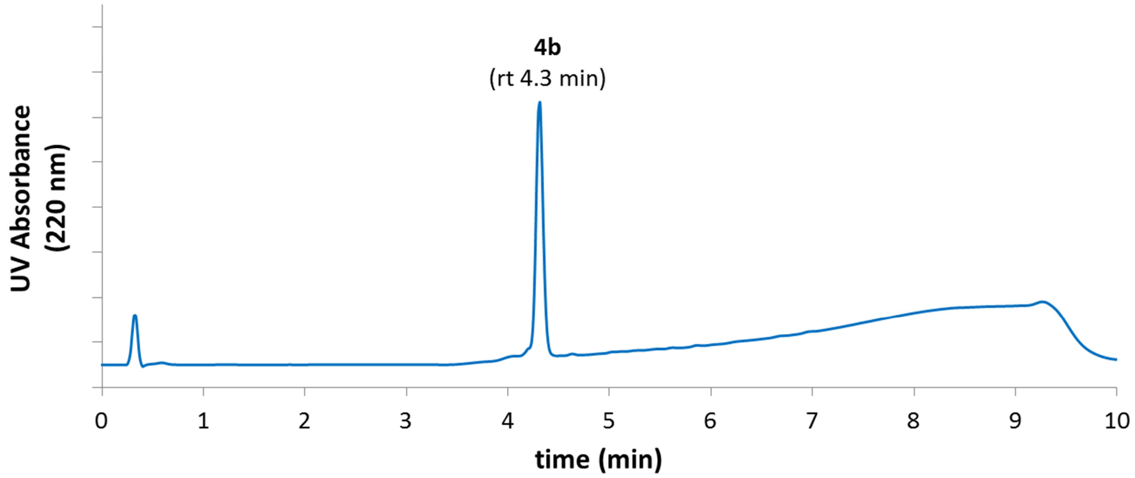

Supplementary Figure 34. UPLC-UV trace of pure peptide **4b** after purification by semi-preparative HPLC using a TFA gradient.

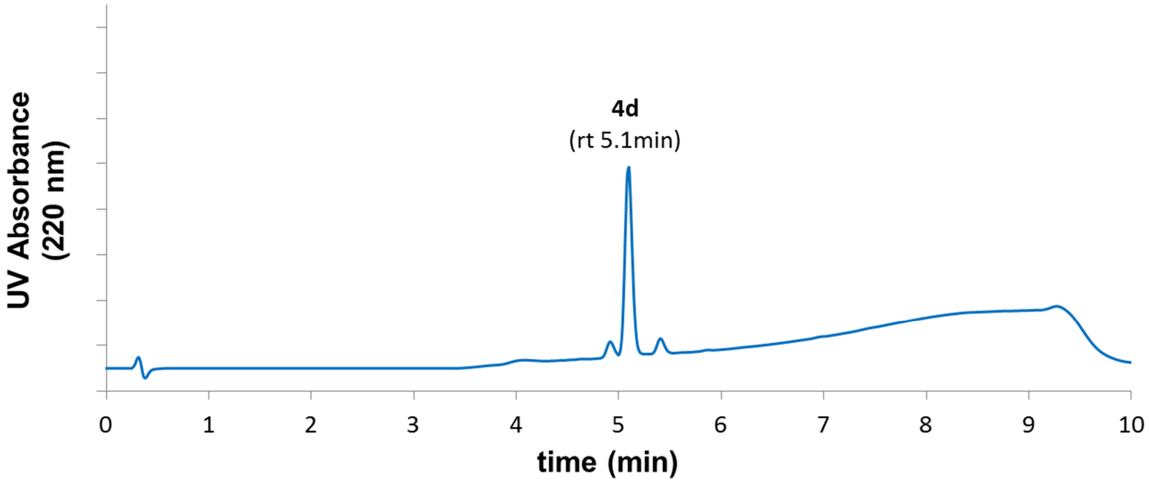

Supplementary Figure 35. UPLC-UV trace of pure peptide **4d** after purification by semi-preparative HPLC using a TFA gradient.

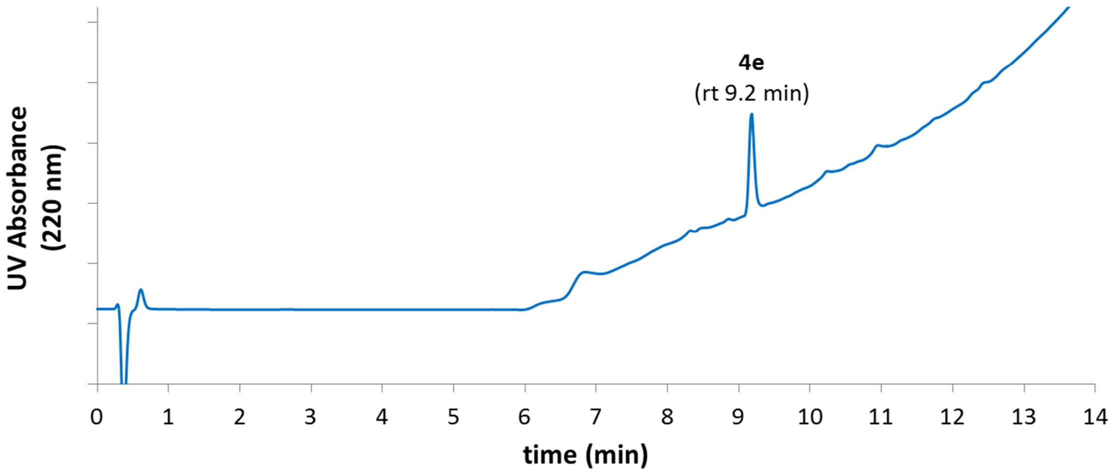

Supplementary Figure 36. UPLC-UV trace of pure peptide **4e** after purification by semi-preparative HPLC using a TFA gradient.

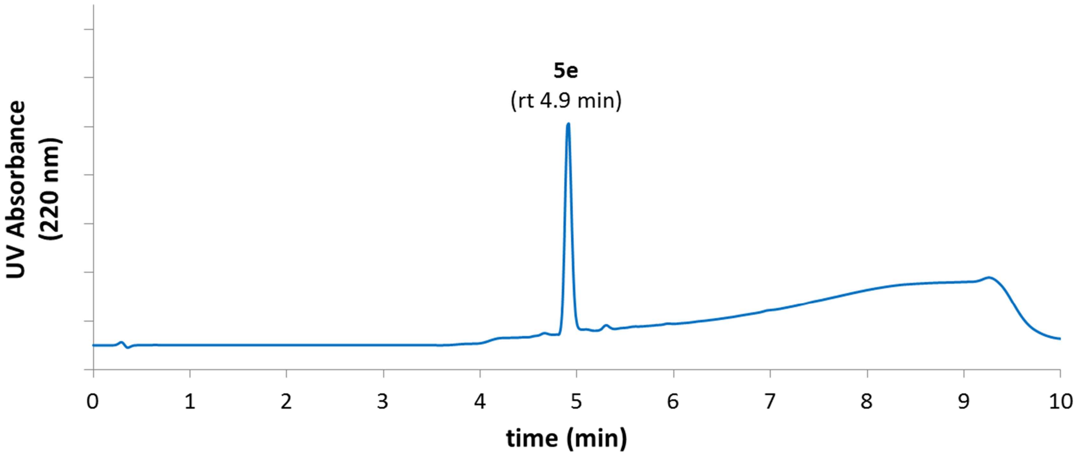

Supplementary Figure 37. UPLC-UV trace of pure peptide **5e** after purification by semi-preparative HPLC using a TFA gradient.

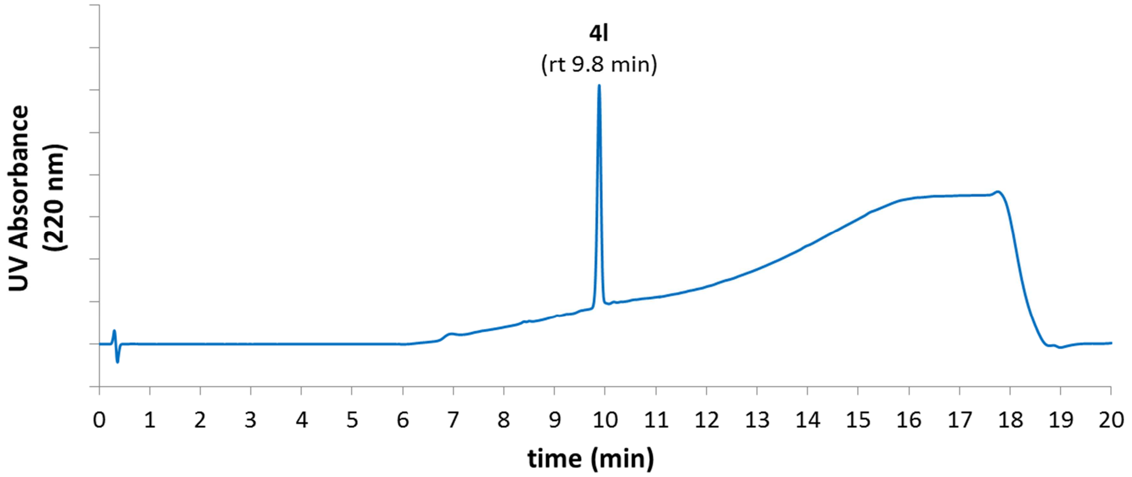

Supplementary Figure 38. UPLC-UV trace of pure peptide **4l** after purification by semi-preparative HPLC using a TFA gradient.

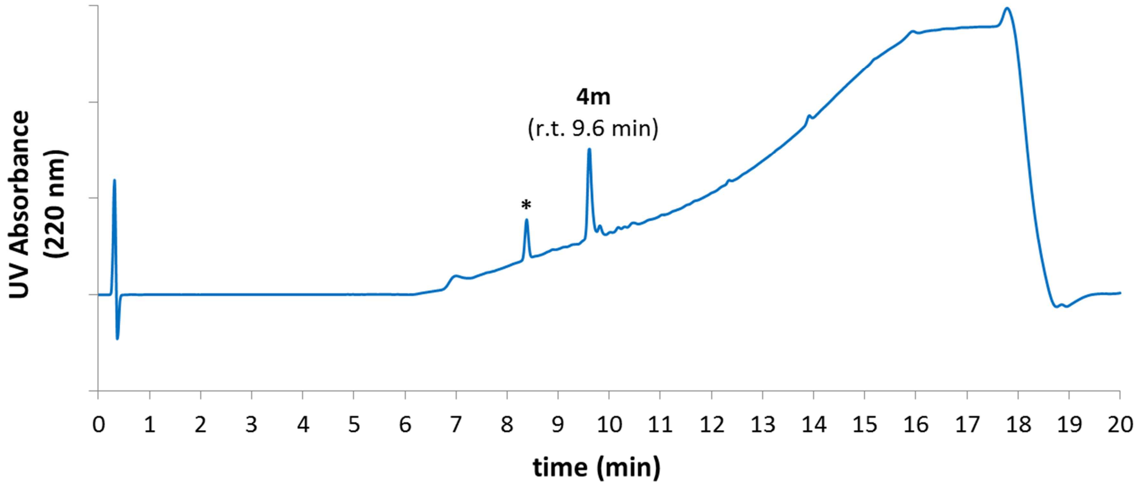

Supplementary Figure 39. UPLC-UV trace of peptide **4m** after purification by semi-preparative HPLC using a TFA gradient. *Side product detected by UPLC-UV at 8.3 min retention time showing no ionization by MS.

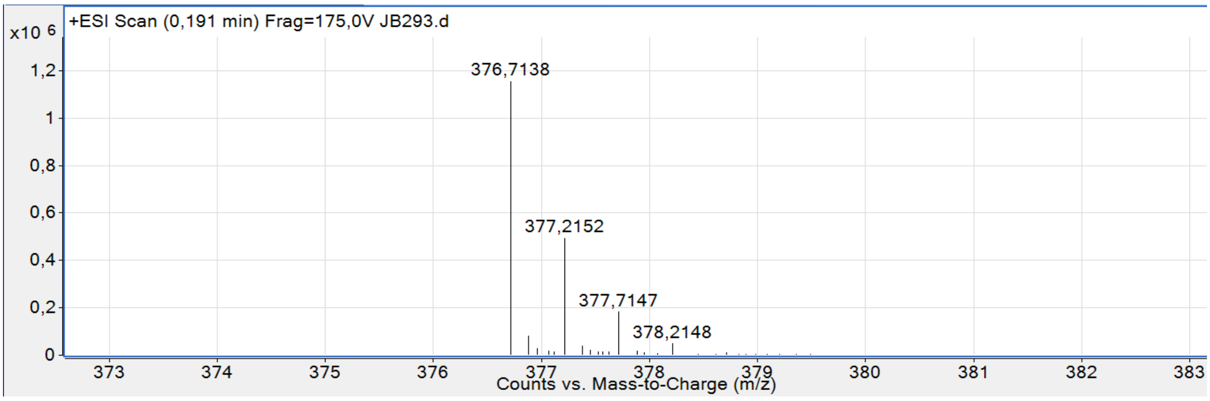

Supplementary Figure 40. Characterization by ESI-MS of Cys-containing peptide 1a. m/z = 376.7138 fragment ion for the $[M+2H]^{2+}$ ion of peptide **1a**.

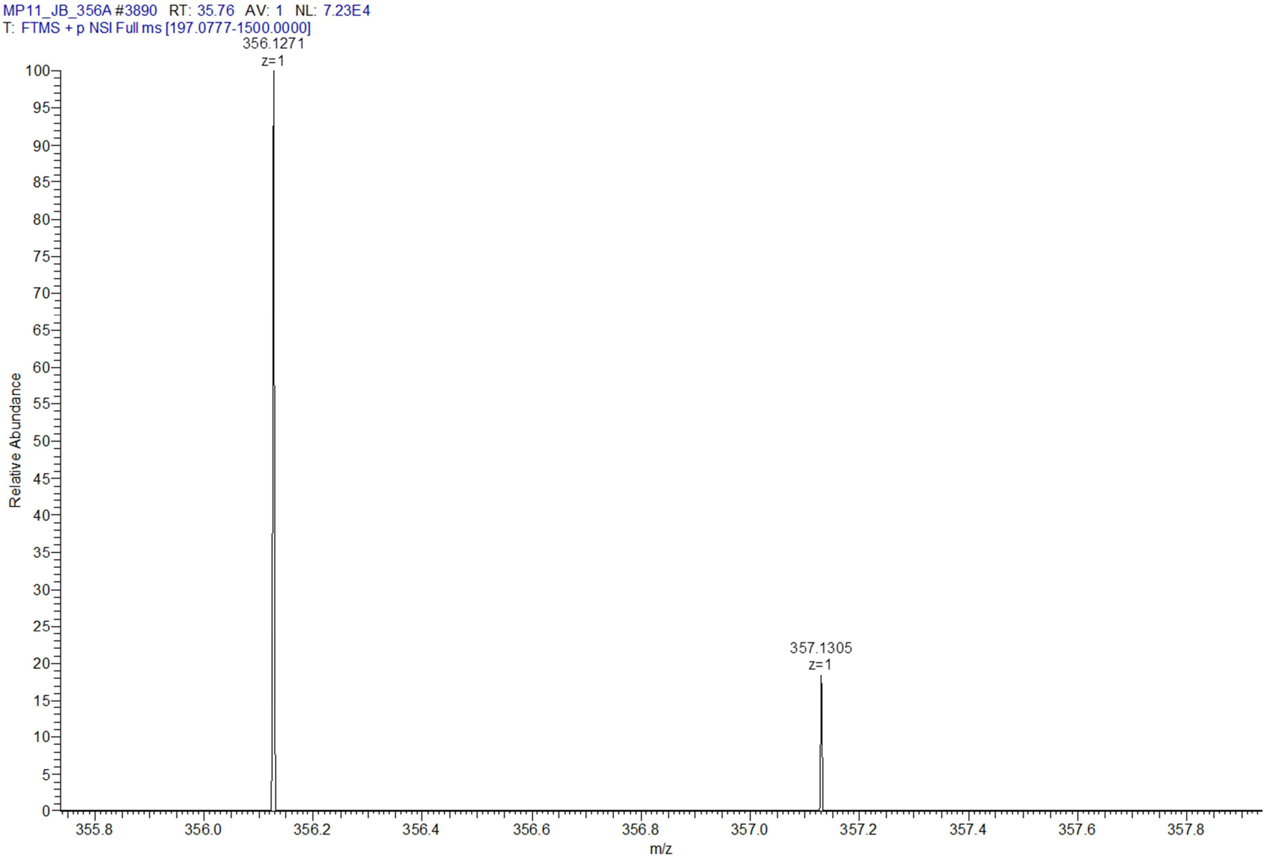

Supplementary Figure 41. Characterization by ESI-MS of Cys-containing peptide 1b and 1c. m/z = 356.1271 fragment ion for the $[M+H]^{+}$ ion of peptide **1b** and **1c**.

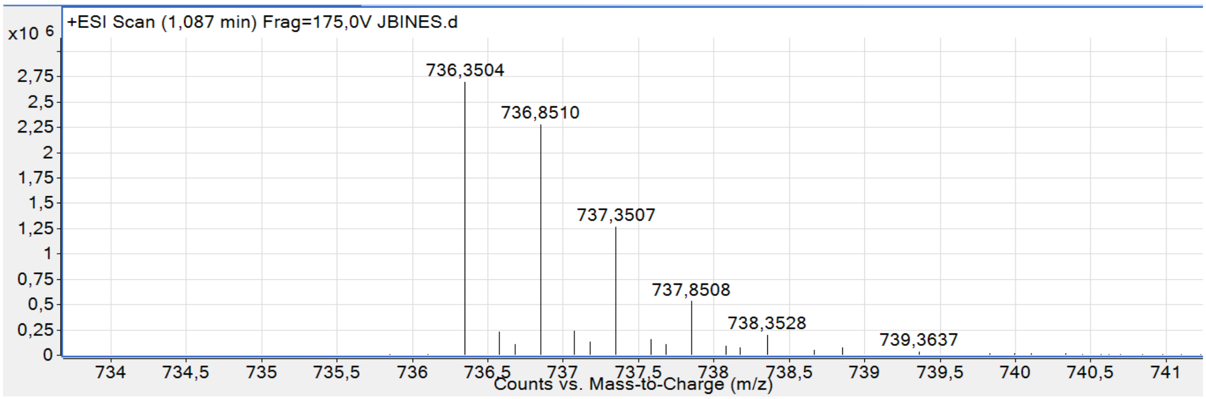

Supplementary Figure 42. Characterization by ESI-MS of Cys-containing peptide 1d. m/z = 736.3504 fragment ion for the $[M+2H]^{2+}$ ion of peptide **1d**.

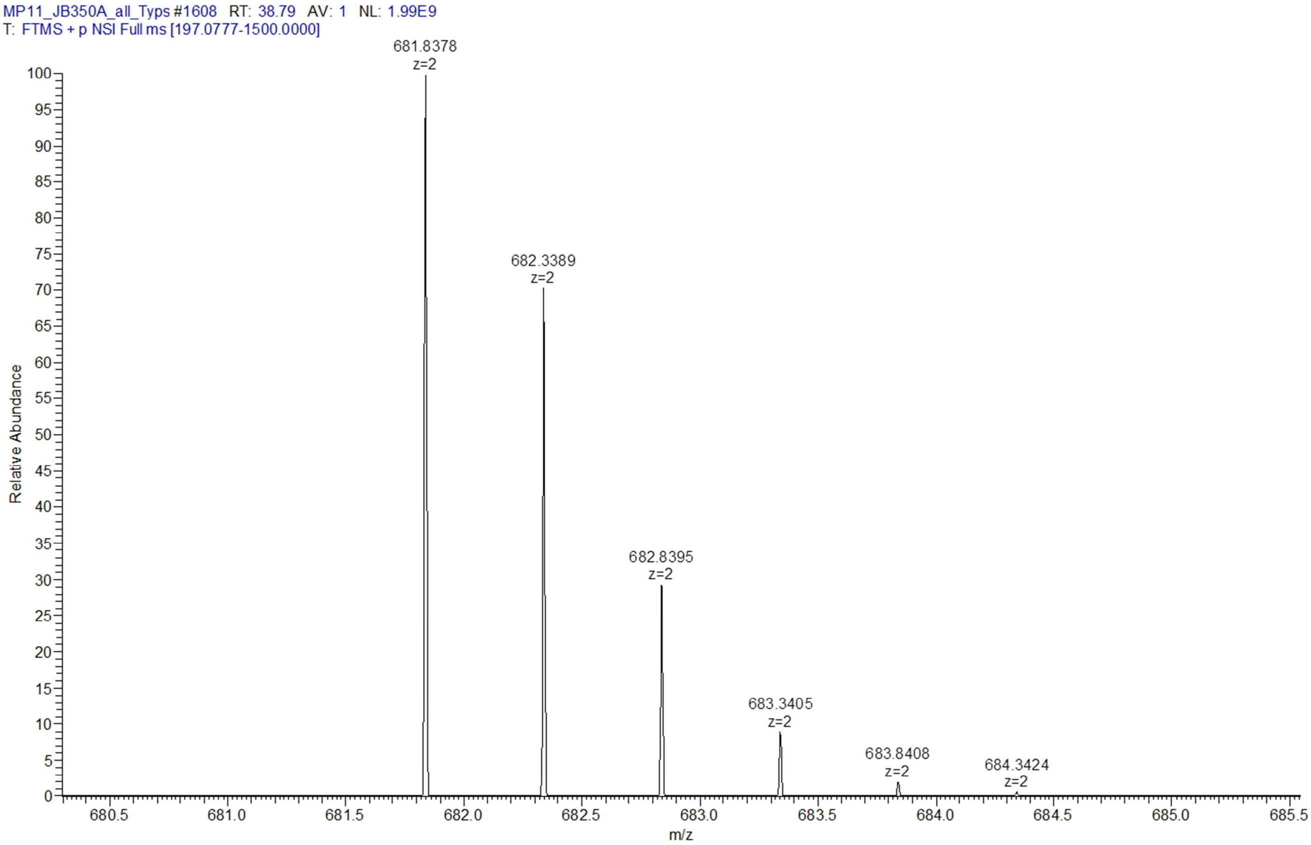

Supplementary Figure 43. Characterization by ESI-MS of Cys-containing peptide 1e. m/z = 681.8378 fragment ion for the $[M+2H]^{2+}$ ion of peptide **1e**.

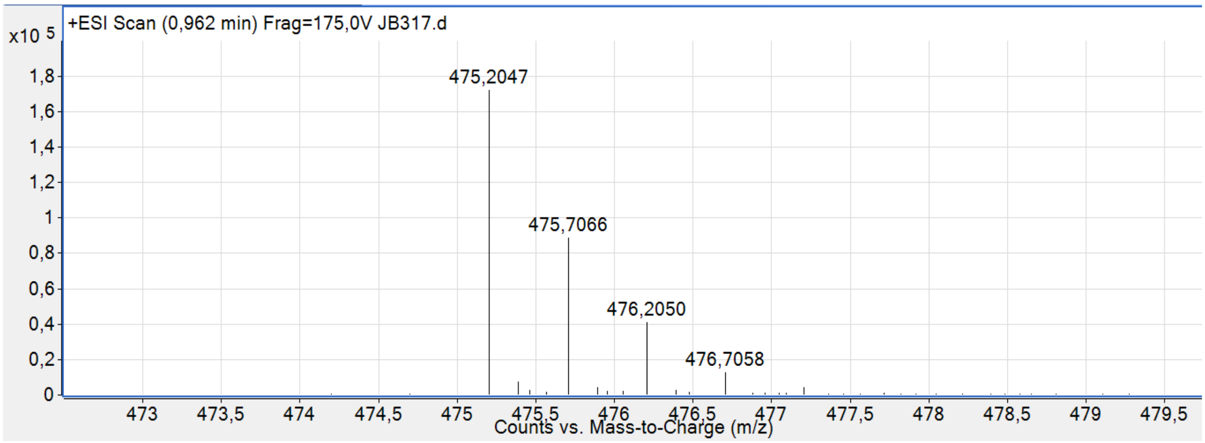

Supplementary Figure 44. Characterization by ESI-MS of Ellman-disulfide peptide 2a. m/z = 475.2047 fragment ion for the $[M+2H]^{2+}$ ion of peptide **2a**.

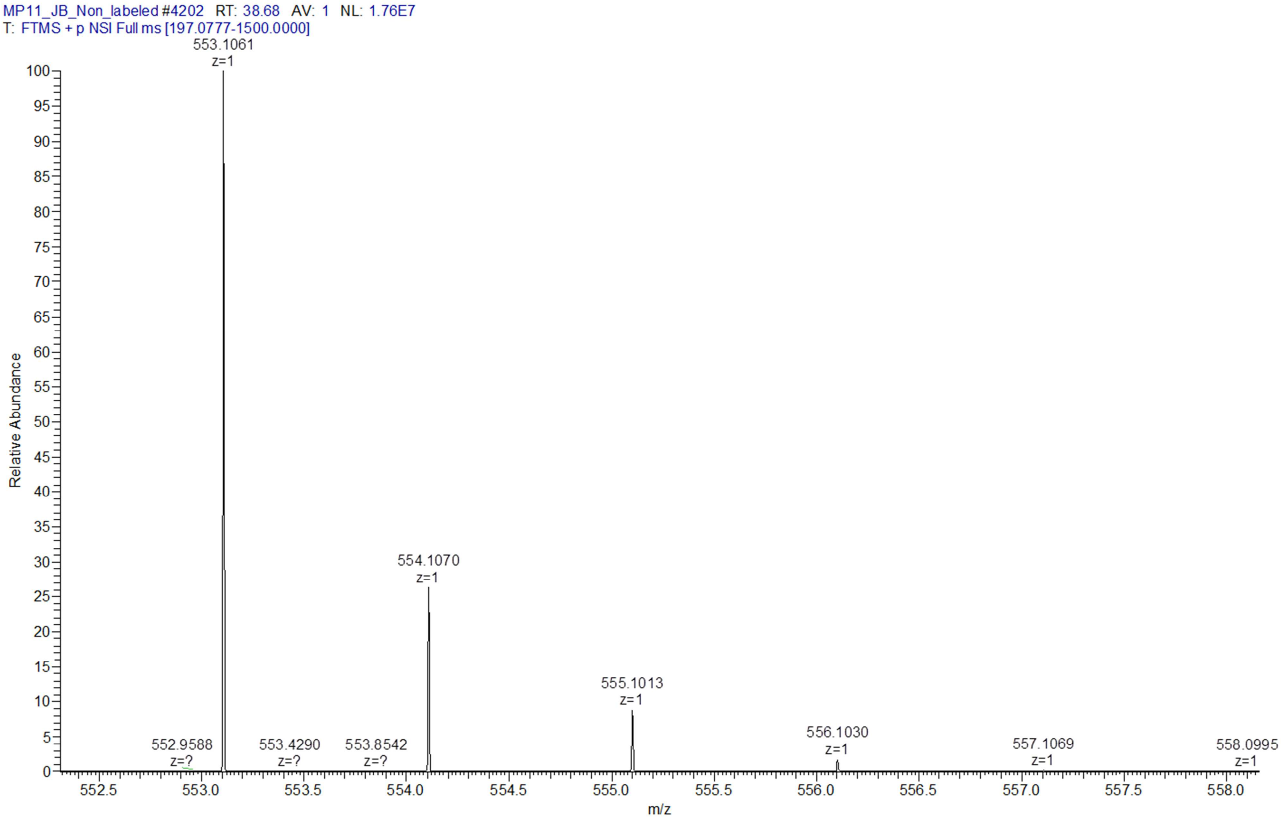

Supplementary Figure 45. Characterization by ESI-MS of Ellman-disulfide peptide 2b and 2c. m/z = 553.1061 fragment ion for the $[M+H]^{+}$ ion of peptide **2b** and **2c**.

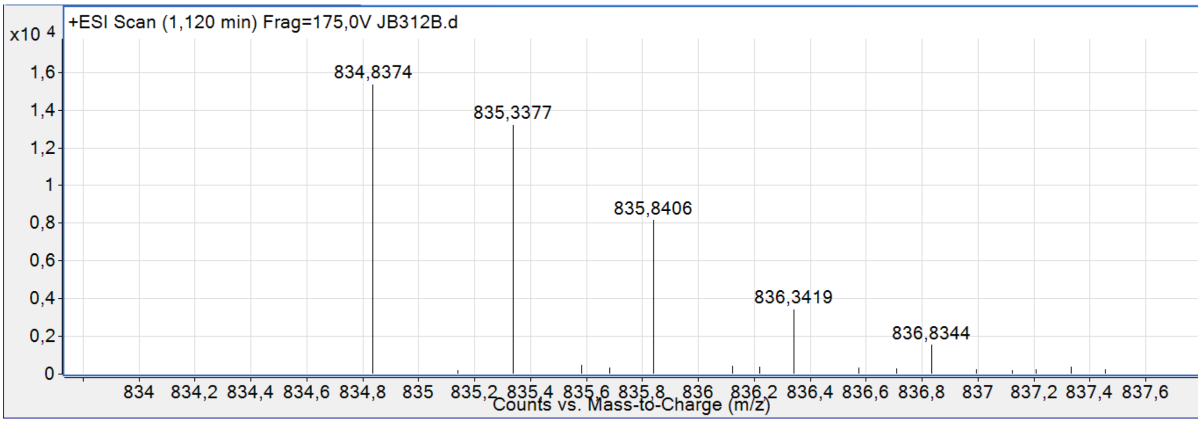

Supplementary Figure 46. Characterization by ESI-MS of Ellman-disulfide peptide 2d. m/z = 834.8374 fragment ion for the $[M+2H]^{2+}$ ion of peptide **2d**.

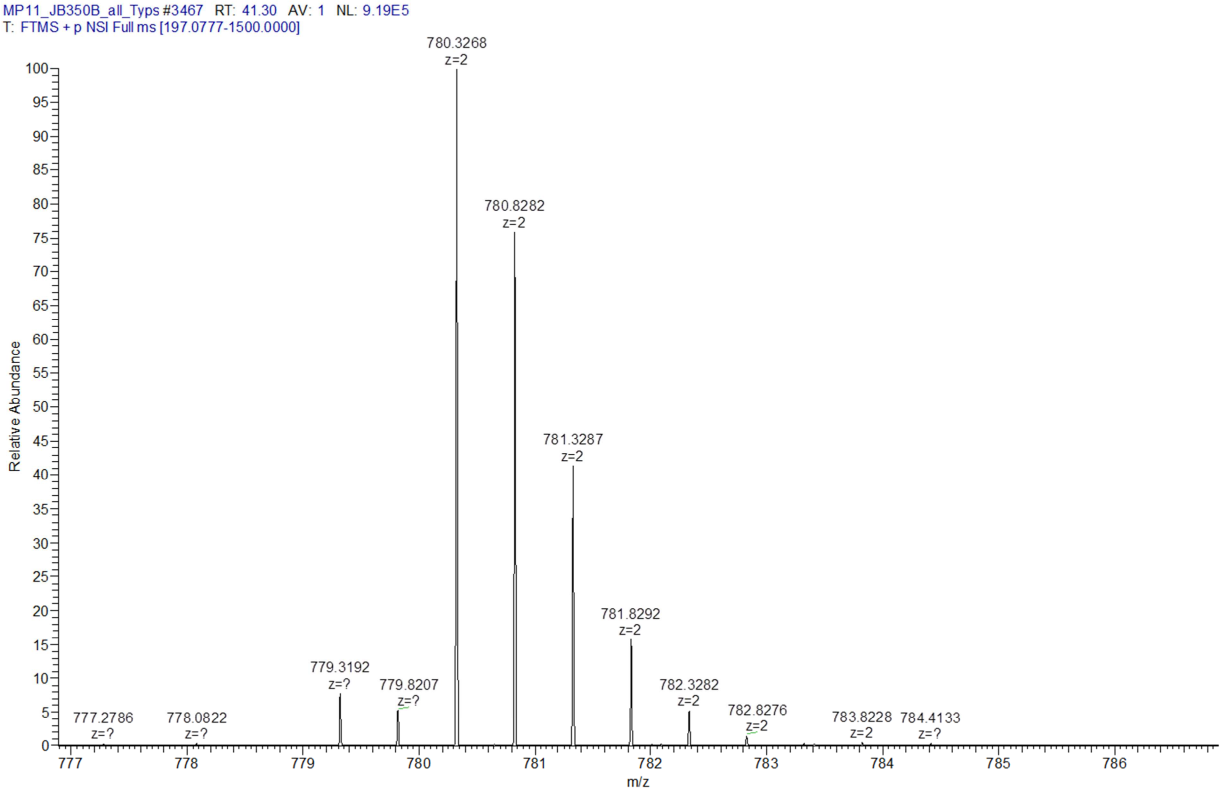

Supplementary Figure 47. Characterization by ESI-MS of Ellman-disulfide peptide 2e. m/z = 780.3268 fragment ion for the $[M+2H]^{2+}$ ion of peptide **2e**.

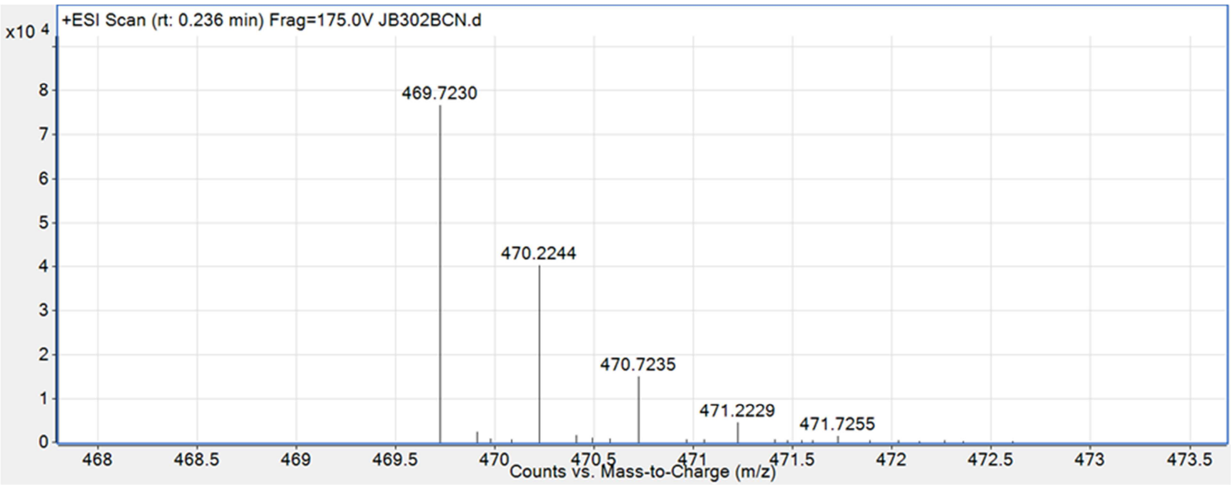

Supplementary Figure 48. Characterization by ESI-MS of *O,O*-bis(2-cyanoethyl) S-cysteine phosphorothiolate peptide 4b. m/z = 469.7230 fragment ion for the $[M+2H]^{2+}$ ion of peptide **4b.**

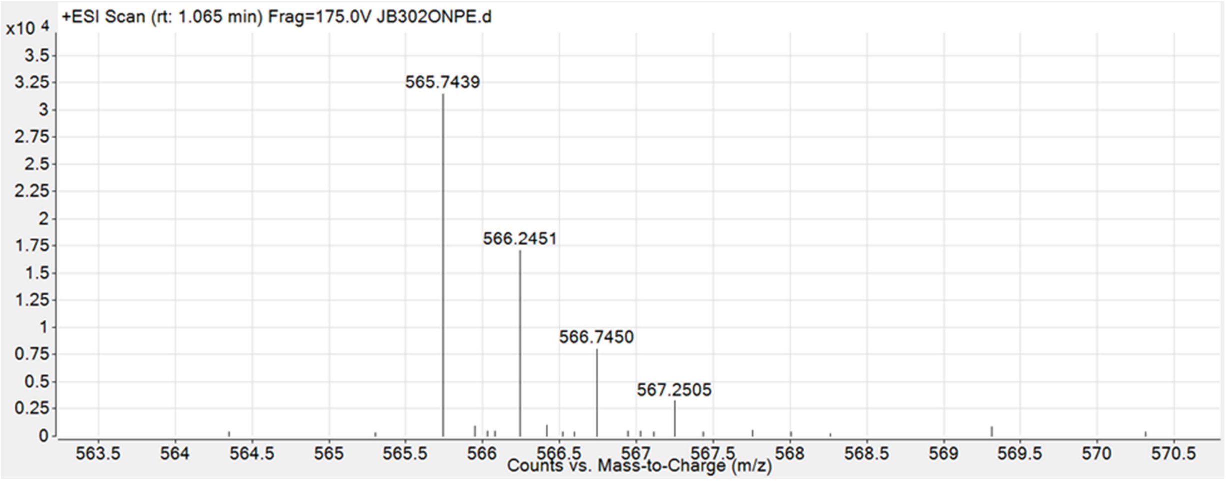

Supplementary Figure 49. Characterization by ESI-MS of *O,O*-bis(1-(2-nitrophenyl)ethyl) *S*-cysteine phosphorothiolate peptide 4d. m/z = 565.7439 fragment ion for the $[M+2H]^{2+}$ ion of peptide **4d.**

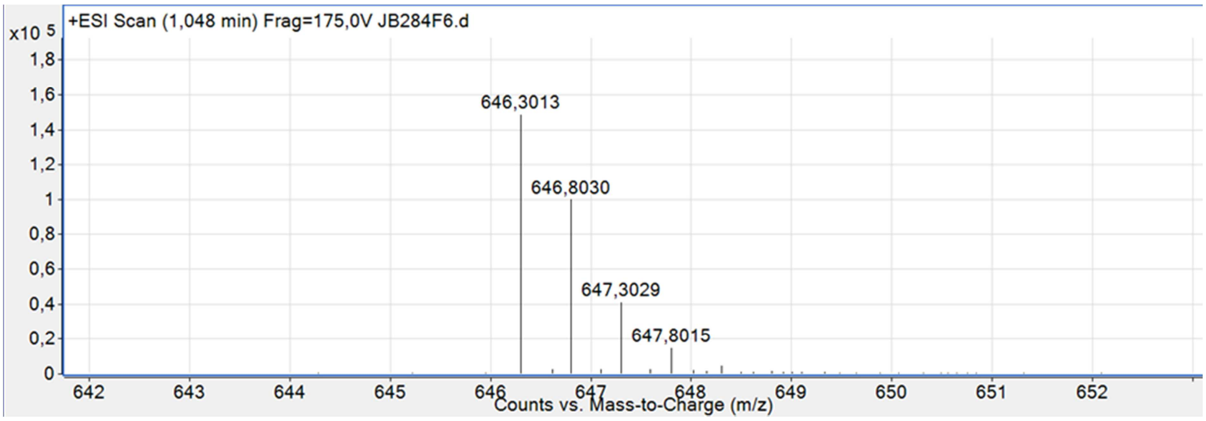

Supplementary Figure 50. Characterization by ESI-MS of *O*-(4-((2,5,8,11,14,17-hexaoxanonadecan-19-yl)oxy)-5-methoxy-2-nitrobenzyl) *S*-cysteine phosphoro-thiolate peptide 5e. m/z = 646.3013 fragment ion for the $[M+2H]^{2+}$ ion of peptide **5e.**

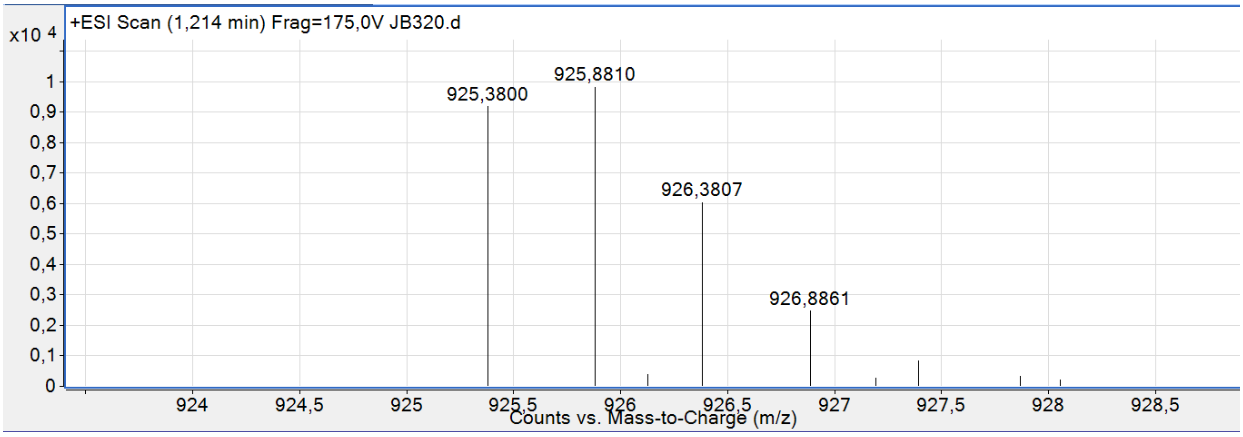

Supplementary Figure 51. Characterization by ESI-MS of *O,O*-bis(1-(2-nitrophenyl)ethyl) *S*-cysteine phosphorothiolate peptide 4l. m/z = 925.3800 fragment ion for the $[M+2H]^{2+}$ ion of peptide **4l.**

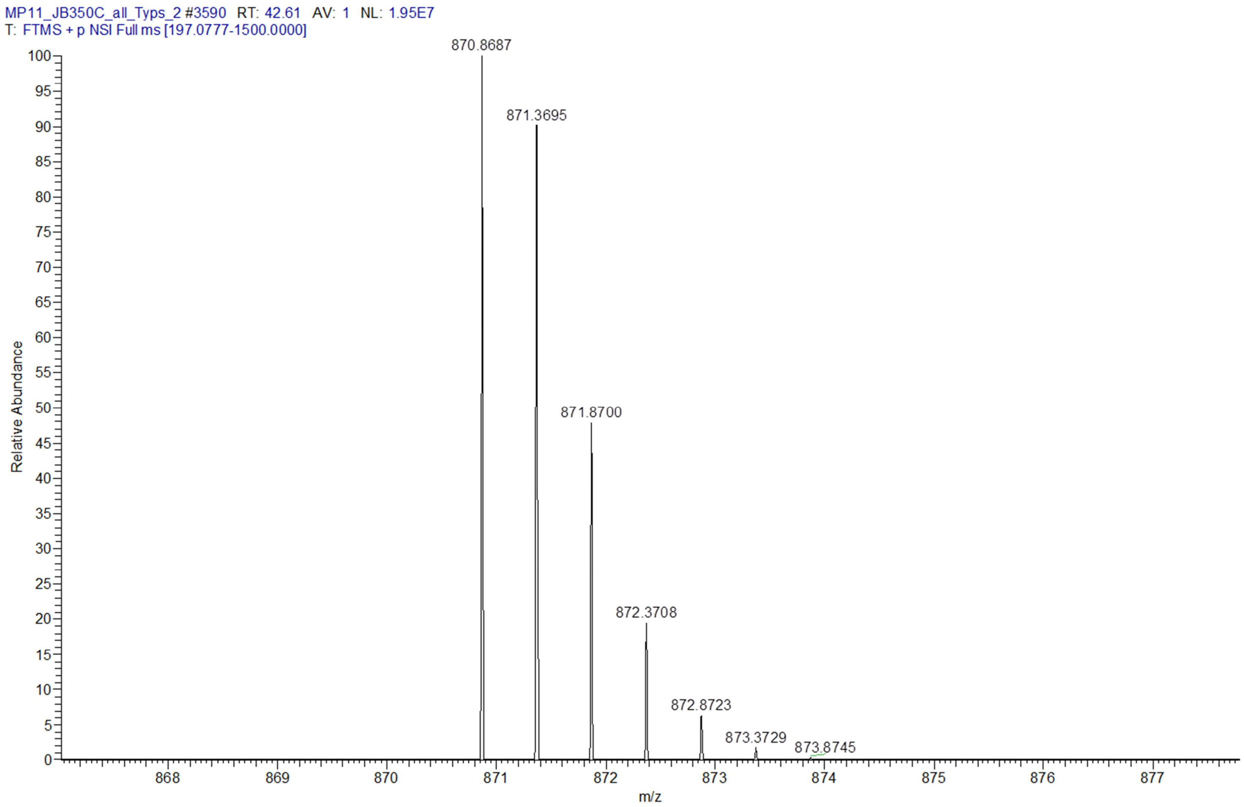

Supplementary Figure 52. Characterization by ESI-MS of *O,O*-bis(1-(2-nitrophenyl)ethyl) *S*-cysteine phosphorothiolate peptide 4m. m/z = 870.8687 fragment ion for the $[M+2H]^{2+}$ ion of peptide **4m.**

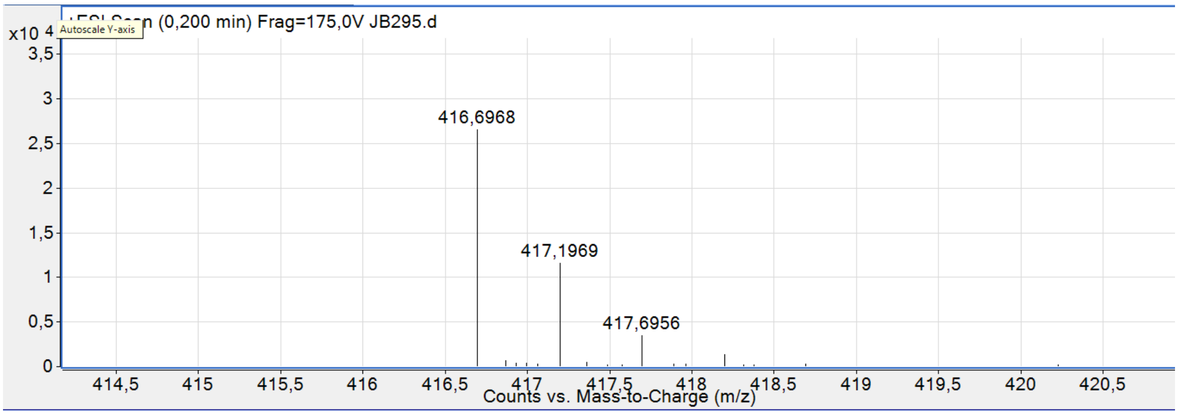

Supplementary Figure 53. Characterization by ESI-MS of phosphorylated Cys peptide 6a. m/z = 416.6968 fragment ion for the $[M+2H]^{2+}$ ion of peptide **6a.**

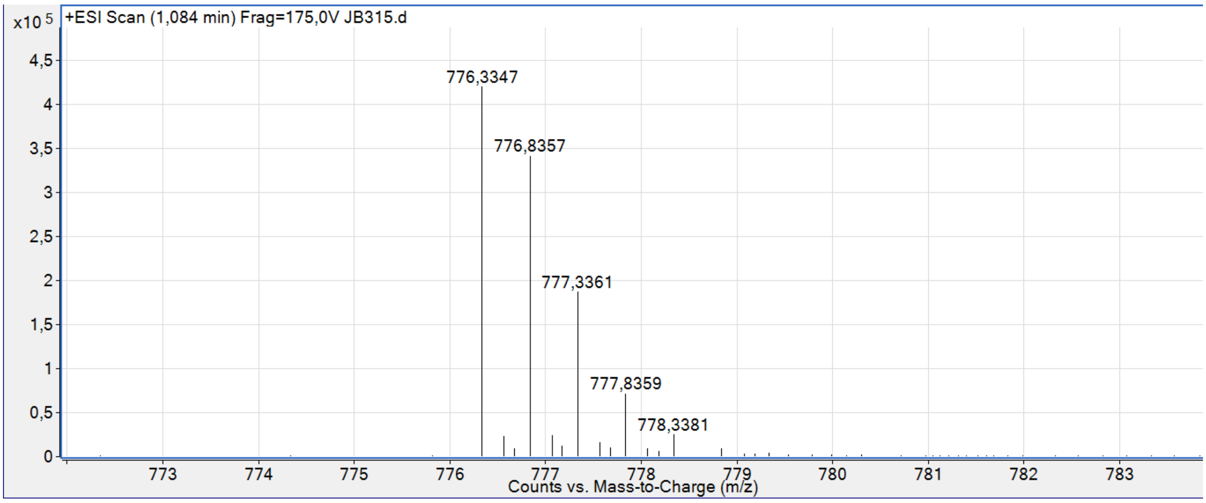

Supplementary Figure 54. Characterization by ESI-MS of phosphorylated Cys peptide 6b. m/z = 776.3347 fragment ion for the $[M+2H]^{2+}$ ion of peptide **6b.**

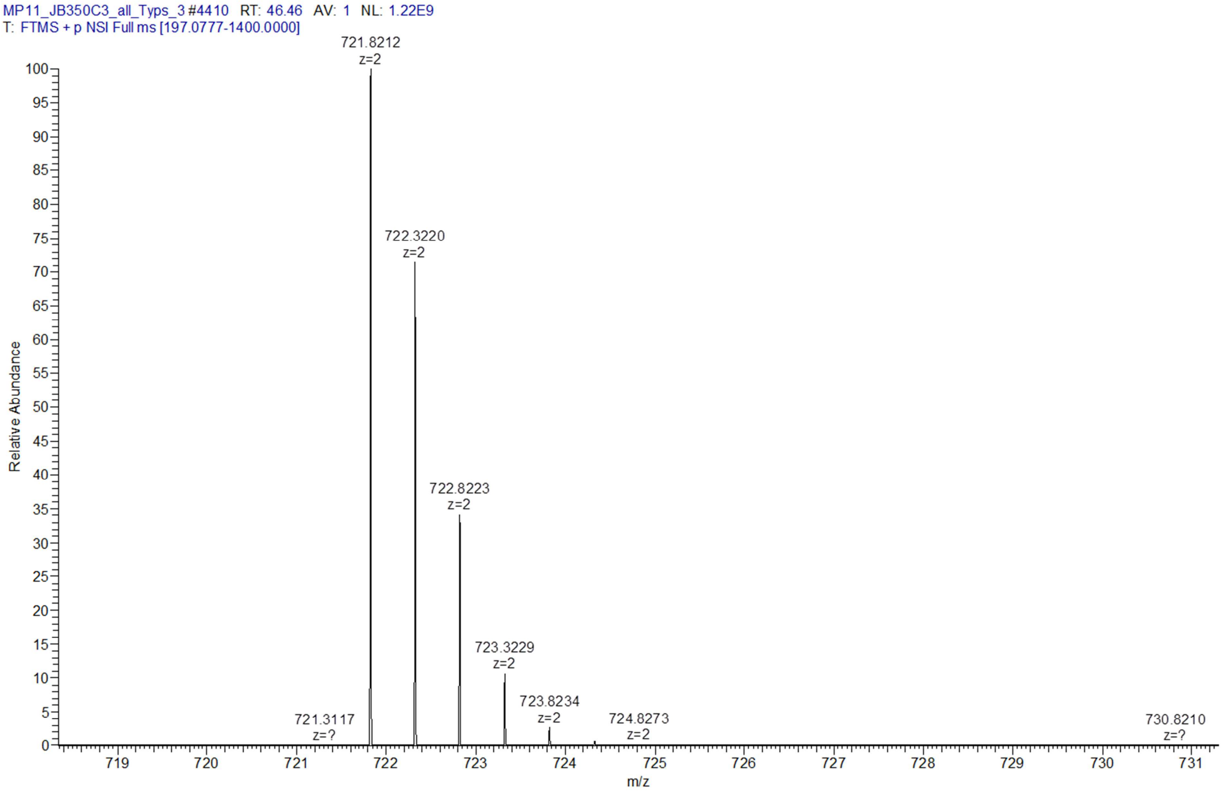

Supplementary Figure 55. Characterization by ESI-MS of phosphorylated Cys peptide 6c. m/z = 721.8212 fragment ion for the $[M+2H]^{2+}$ ion of peptide **6c.**

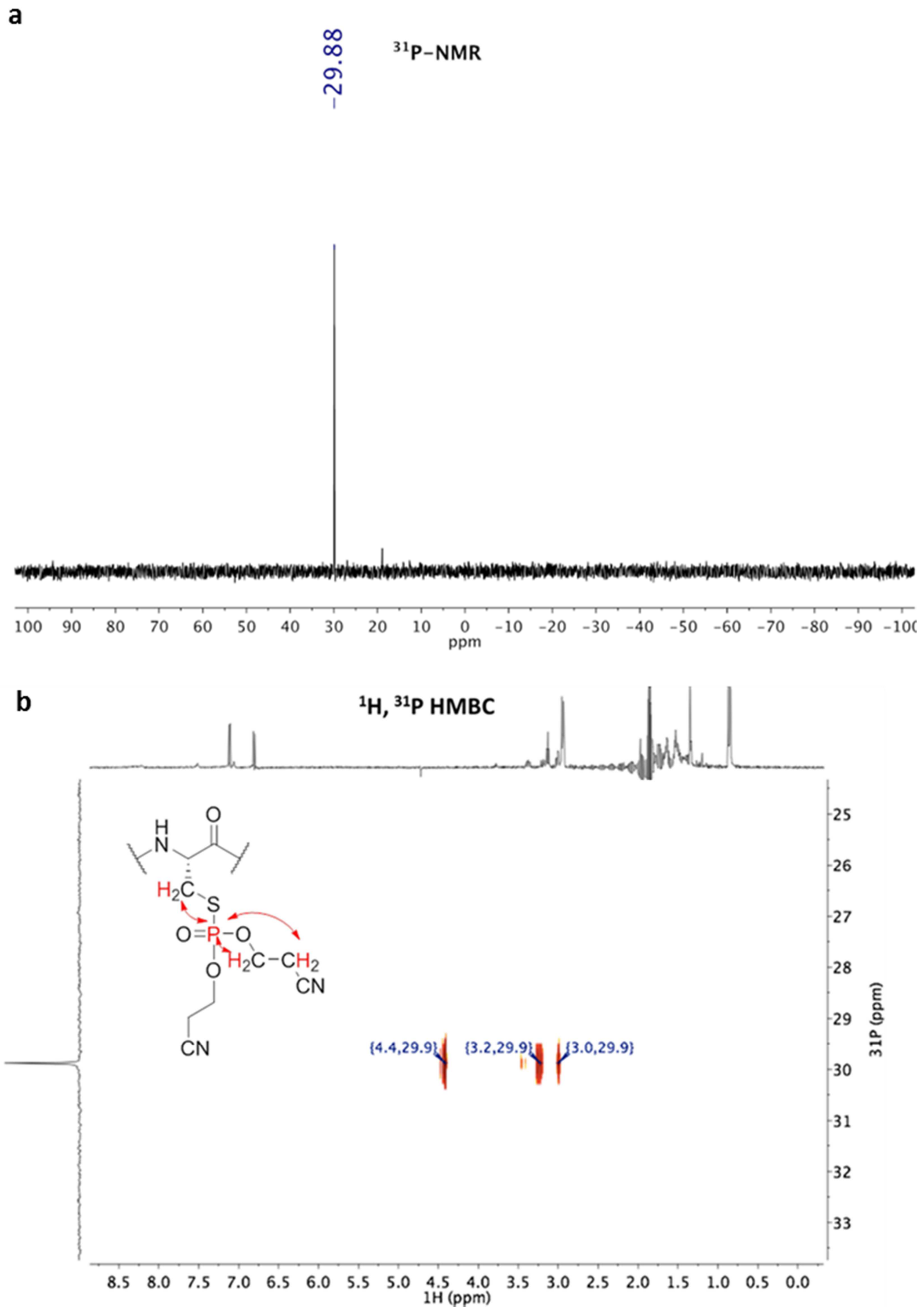

Supplementary Figure 56. Characterization by NMR of *O,O*-bis(2-cyanoethyl) *S*-cysteine phosphorothiolate peptide 4b. (**a**) ^{31}P NMR and (**b**) ^{1}H, ^{31}P HMBC NMR spectra of phosphorothiolate ester peptide **4b.** The phosphorous signal peak at 29.9 ppm shows coupling to α-methylene hydrogen atoms of the pCys side chain (at 3.2 ppm) and to the hydrogen atoms of the 2-cyanoethyl protecting groups (at 4.4 ppm and at 3.0 ppm).

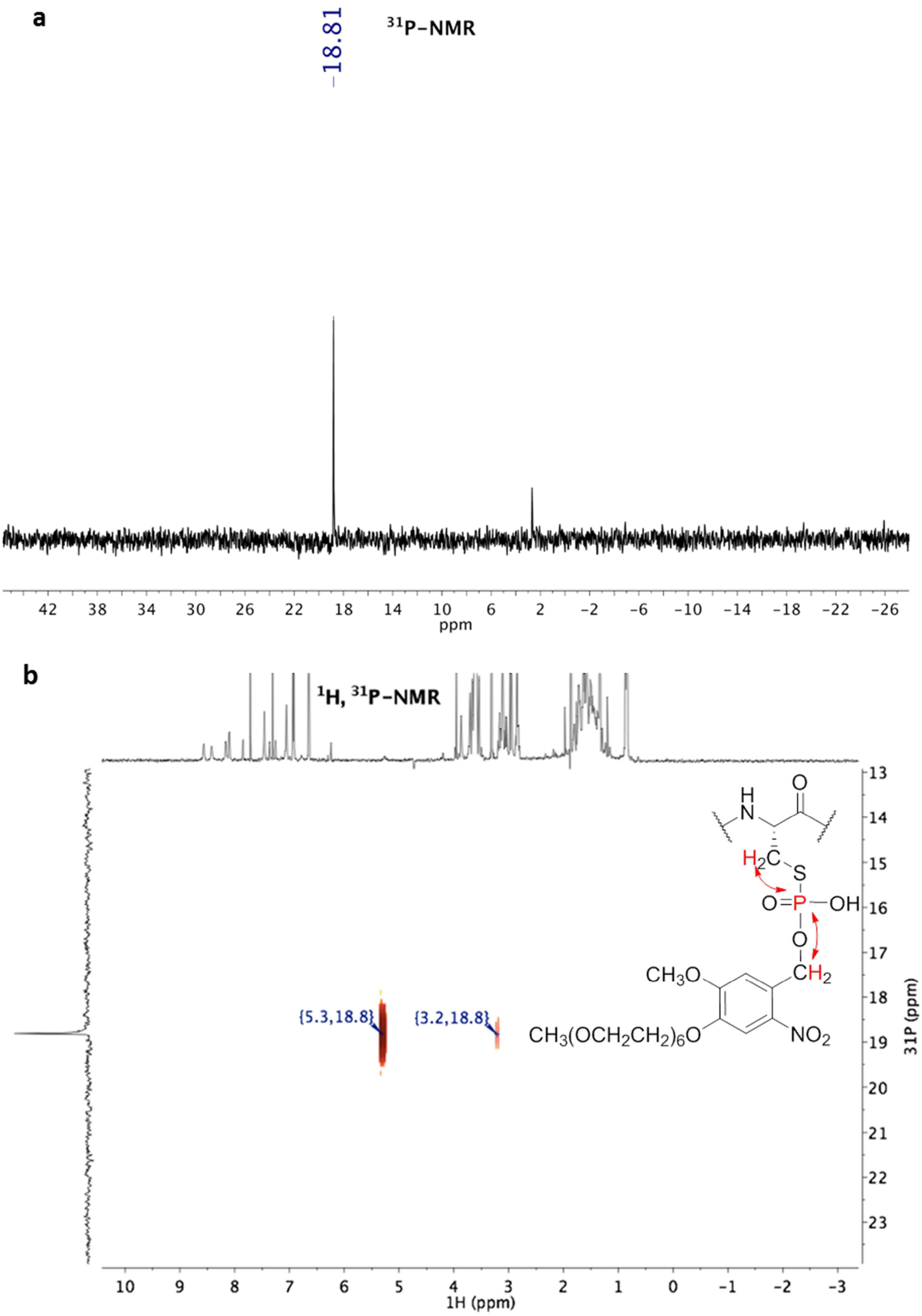

Supplementary Figure 57. Characterization by NMR of O-(4-((2,5,8,11,14,17-hexaoxanonadecan-19-yl)oxy)-5-methoxy-2-nitrobenzyl)S-cysteine phosphoro-thiolate peptide 5e. (**a**) ^{31}P NMR and (**b**) ^{1}H, ^{31}P HMBC NMR spectrum of phosphorothiolate ester peptide **5e.** The phosphorous signal peak at 18.8 ppm shows coupling to α-methylene hydrogen atoms of the pCys side chain (at 3.2 ppm) and to the hydrogen atoms of the *o*-nitrobenzyl protecting group (at 5.3 ppm).

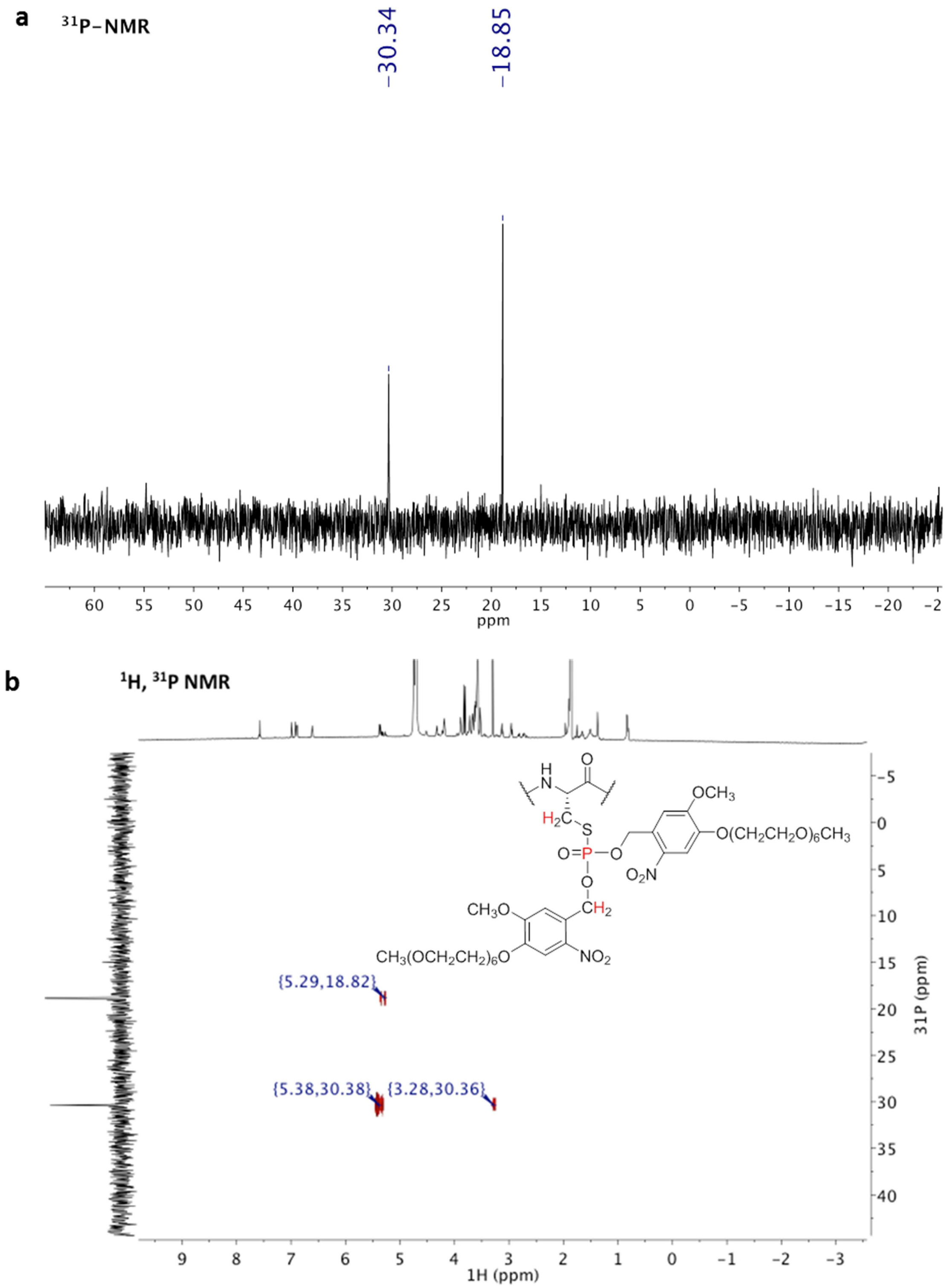

Supplementary Figure 58. Characterization by NMR of O,O-bis((4-((2,5,8,11,14,17-hexaoxanonadecan-19-yl)oxy)-5-methoxy-2-nitrobenzyl) S-cysteine phosphorothiolate peptide 4e. (**a**) ^{31}P NMR and (**b**) ^{1}H, ^{31}P HMBC NMR spectrum of phosphorothiolate ester peptide **4e.** The phosphorous signal peak at 30.34 ppm shows coupling to α-methylene hydrogen atoms of the pCys side chain (at 3.28 ppm) and to the hydrogen atoms of the *o*-nitrobenzyl protecting group (at 5.38 ppm).

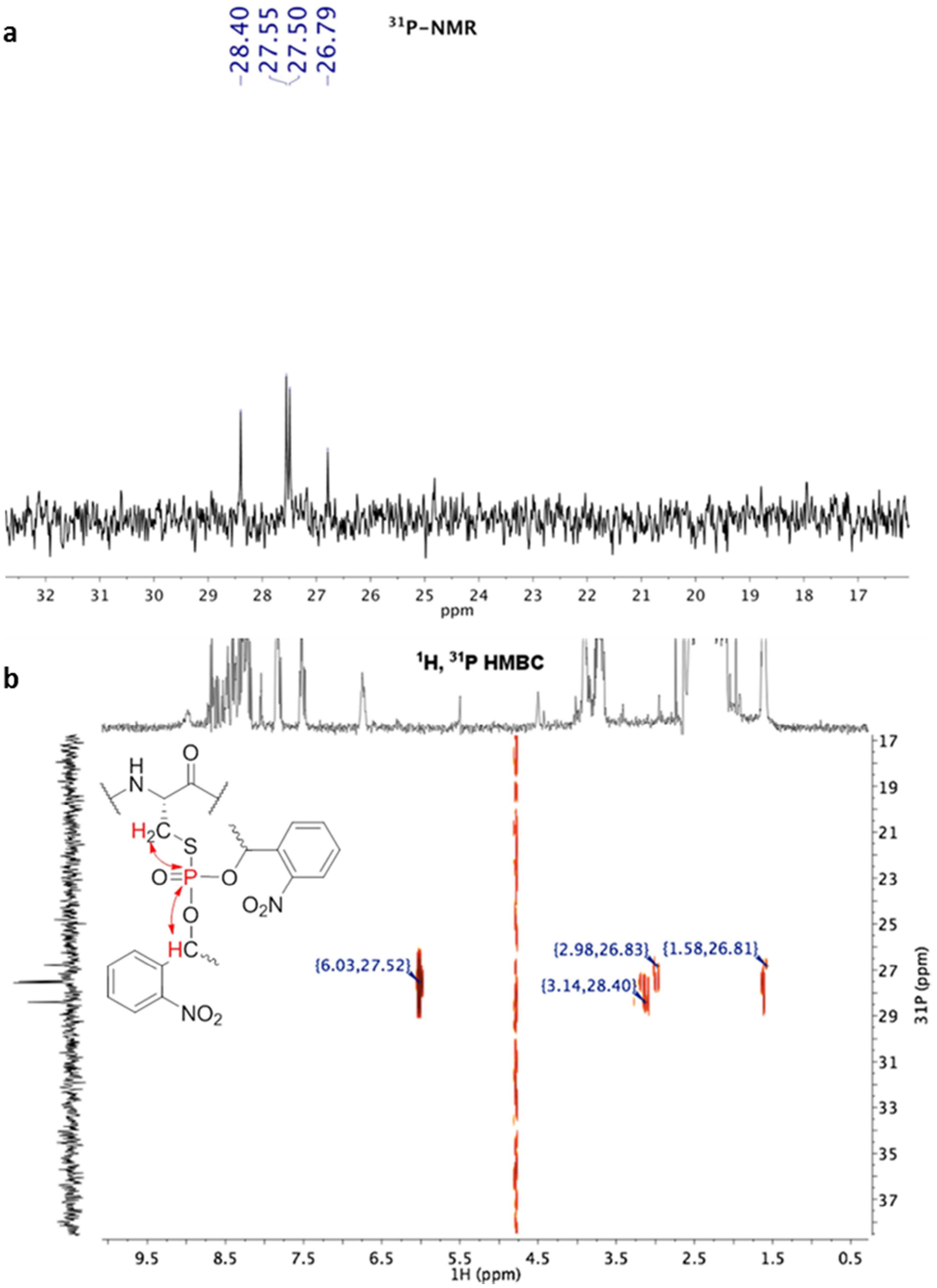

Supplementary Figure 59. Characterization by NMR of O,O-bis(1-(2-nitrophenyl)ethyl) S-cysteine phosphorothiolate peptide 4d. (**a**) ^{31}P NMR and (**b**) ^{1}H, ^{31}P HMBC NMR spectrum of phosphorothiolate ester peptide **4d**. The phosphorous signal peaks at 28.40, 27.55 ppm, 27.50 ppm, and 26.79 ppm shows coupling to α-methylene hydrogen atoms from the pCys side chain (at 3.14 – 2.98 ppm), with the methine hydrogen at the benzylic position (at 6.03 ppm) and with the methyl hydrogens (1.58 ppm) of the *o*-nitrobenzyl protecting group.

HCD MS/MS spectra

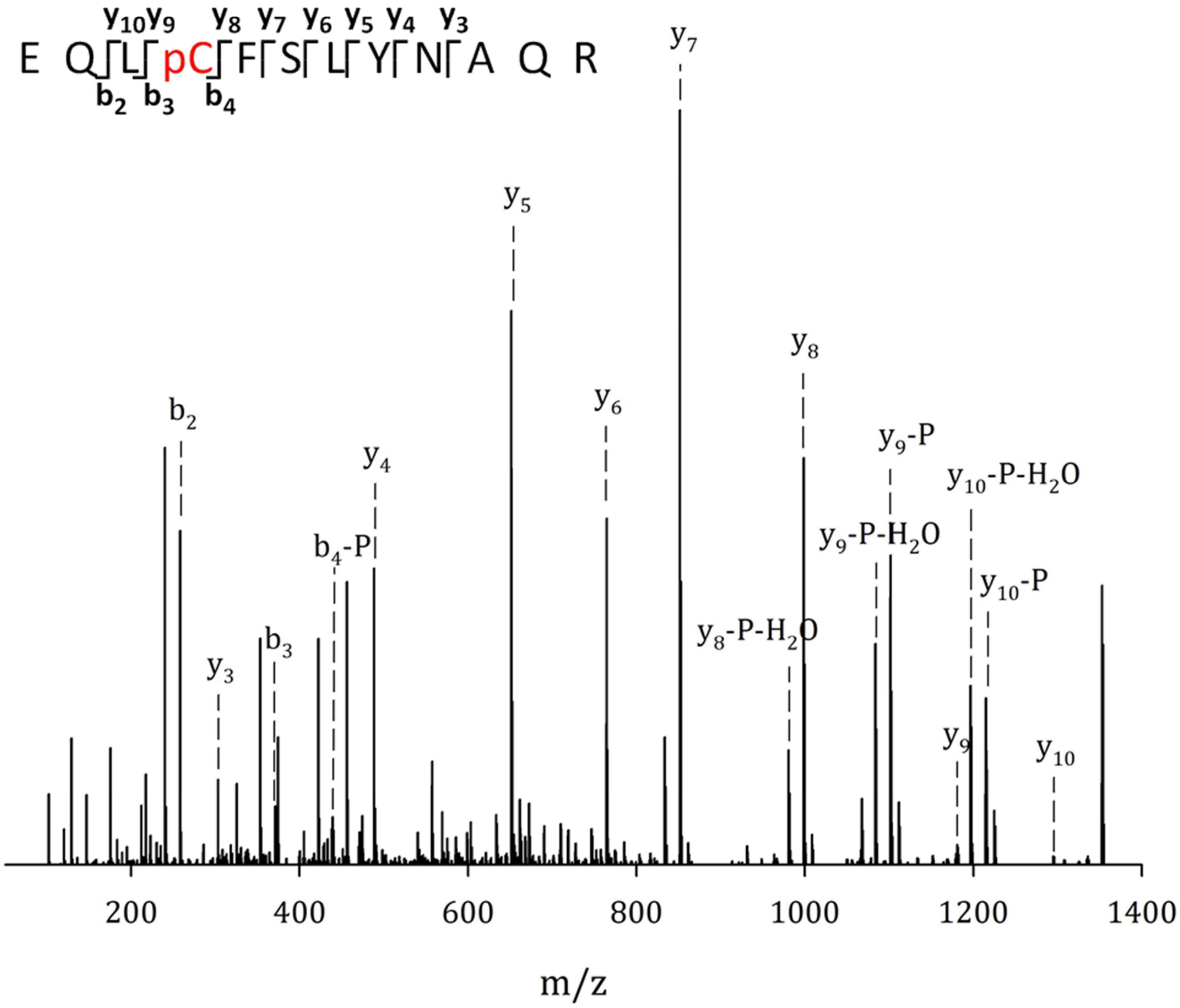

Supplementary Figure 60. Characterization by MS of peptide 6b. HCD MS/MS spectra of phosphorylated cysteine peptide **6b** showing unphosphorylated *b*- and *y*-type fragments.

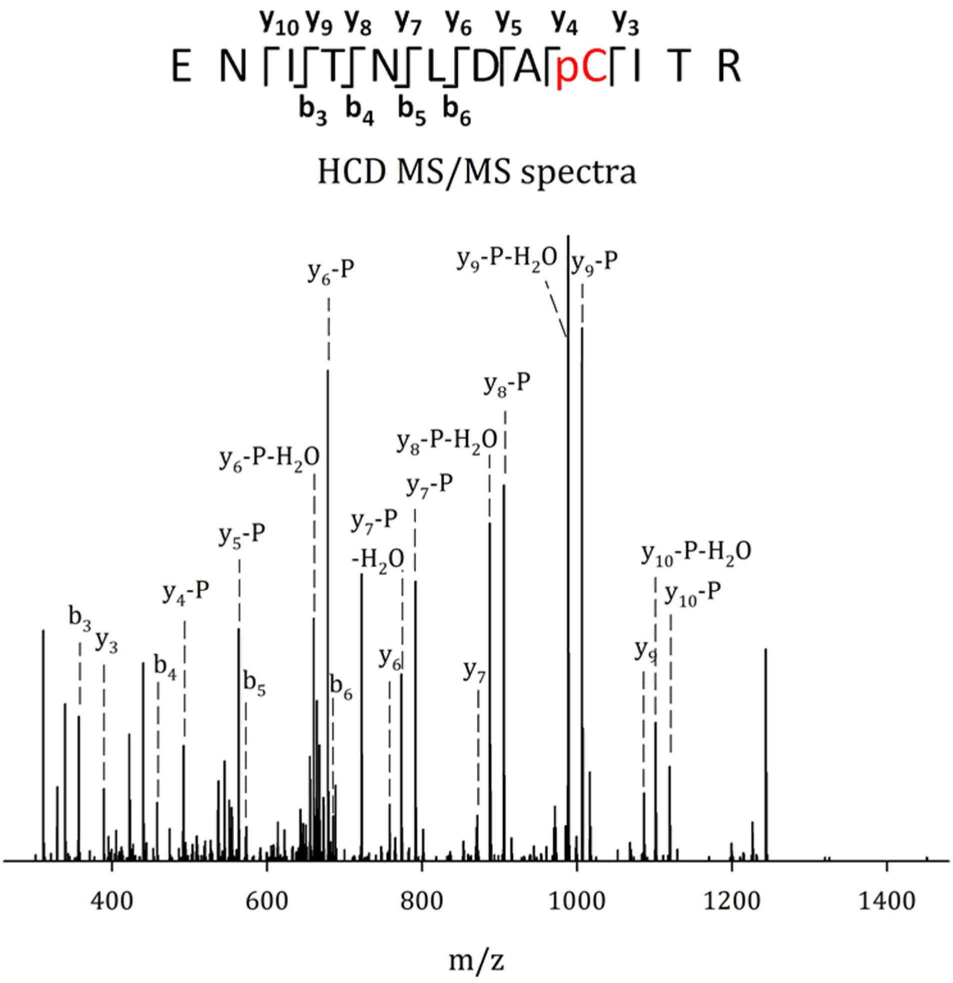

Supplementary Figure 61. Characterization by MS of an endogenous pCys peptide. HCD MS/MS spectra of the endogenous pCys ENITNLDApCITR peptide showing unphosphorylated *b*- and *y*-type fragments.

Supplementary Methods

Synthesis of peptide 1a

Procedure: Rink Amide AM Resin (0.2 mmol; 0.73 mmol/g; 100-200 mesh) was loaded with Fmoc-Lys(Boc)-OH and applied to SPPS using Fmoc-couplings with HOBt/HBTU/DIPEA in DMF on a Tribute Peptide Synthesizer (Protein Technologies, Inc). The peptide was cleaved off the resin by addition of TFA/DTT/TIS/thioanisol (95/2/2/1), followed by precipitation of the peptide in cold ether. Peptide **1a** was used without further purification. ESI-MS (positive mode) = 376.7138 $[M+2H]^{2+}$ (calcd. m/z: 376.7154).

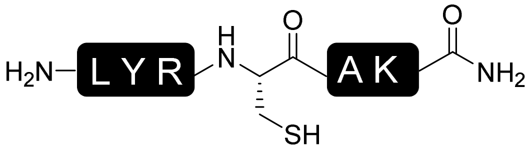

1a

Synthesis of peptide 1b

Procedure: A Wang resin (0.2 mmol; 0.71 mmol/g; 100-200 mesh) preloaded with Fmoc-Ala-OH was used and applied to SPPS using Fmoc-coupling with HOBt/HBTU/DIPEA in DMF. The peptide was cleaved off the resin by addition of TFA/DTT/TIS/thioanisol (95/2/2/1), followed by precipitation of the peptide in cold ether. Peptide **1b** was used without further purification. ESI-MS (positive mode) = 356.1271 $[M+H]^{+}$ (calcd. m/z: 356.1275).

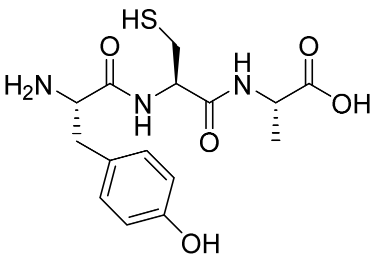

1b

Synthesis of peptide 1c

Procedure: A Wang resin (0.2 mmol; 0.71 mmol/g; 100-200 mesh) preloaded with Fmoc-Ala-OH was used and applied to SPPS using Fmoc-coupling with HOBt/HBTU/DIPEA in DMF. The peptide was cleaved off the resin by addition of TFA/DTT/TIS/thioanisol (95/2/2/1), followed by precipitation of the peptide in cold ether. Peptide **1c** was used without further purification. ESI-MS (positive mode) = 356.1271 $[M+H]^+$ (calcd. m/z: 356.1275).

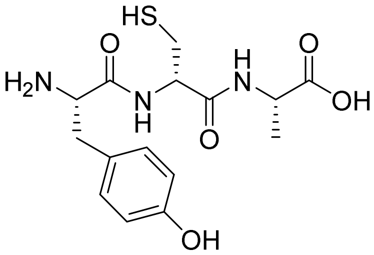

1c

Synthesis of peptide 1d

Procedure: A TentaGel S PHB resin (0.05 mmol; 0.25 mmol/g; 90 mesh) preloaded with Fmoc-Arg(Pbf)-OH was used and applied to SPPS using Fmoc-coupling with HOBt/HBTU/DIPEA in DMF on a automated parallel petide synthesizer Syro II. The peptide was cleaved off the resin by addition of TFA/DTT/TIS/thioanisol (95/2/2/1), followed by precipitation of the peptide in cold ether. Purification *via* semi-preparative HPLC (method A) yielded peptide **1d** (18 mg, 10.6 µmol) in 21% yield. ESI-MS (positive mode) = 736.3504 $[M+2H]^{2+}$ (calcd. m/z: 736.3535).

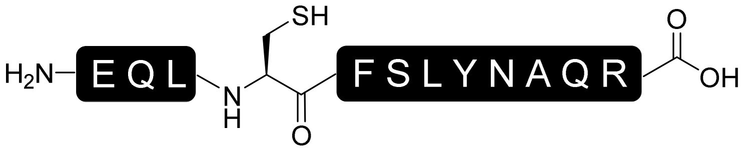

1d

Synthesis of peptide 1e

Procedure: A Wang resin (0.05 mmol; 0.8 mmol/g; 100-200 mesh) preloaded with Fmoc-Arg(Pbf)-OH was used and applied to SPPS using Fmoc-coupling with HOBt/HBTU/DIPEA in DMF. The peptide was cleaved off the resin by addition of TFA/DTT/TIS/thioanisol (95/2/2/1), followed by precipitation of the peptide in cold ether. Purification *via* semi-preparative HPLC (method A) yielded peptide **1e** (16 mg, 11.8 µmol) in 24 % yield. ESI-MS (positive mode) = 681.8378 $[M+2H]^{2+}$ (calcd. m/z: 681.8377).

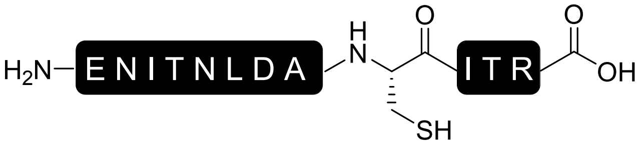

1e

Synthesis of peptide 2a

Procedure: Cysteine peptide **1a** as white trifluoroacetate salt (25 mg, 22.9 µmol) was dissolved in DMF (0.8 mL) along with Et_3N (63.8 µL, 457 µmol, 20 eq). To the stirred solution was added a solution of Ellman´s reagent (18.1 mg, 45.8 µmol, 2 eq) in DMF (0.8 mL). Final peptide concentration was 28.6 mM. The reaction mixture was incubated at room temperature for 15 min. Purification *via* semi-preparative HPLC (method A) yielded peptide **2a** (9.8 mg, 7.6 µmol) in 33% yield. ESI-MS (positive mode) = 475.2047 $[M+2H]^{2+}$ (calcd. m/z: 475.2045).

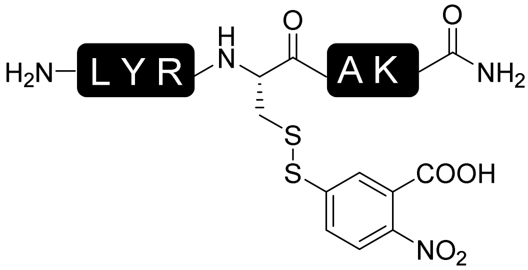

2a

Synthesis of peptide 2b

Procedure: Cysteine peptide **1b** (8.4 mg, 23.6 µmol) was dissolved in DMF (0.35 mL) along with Et_3N (66.0 µL, 472 µmol, 20 eq). To the stirred solution was added a solution of Ellman´s reagent (18.7 mg, 47.2 µmol, 2 eq) in DMF (0.35 mL). Final peptide concentration was 33.7 mM. The reaction mixture was incubated at room temperature for 15 min. Purification *via* semi-preparative HPLC (method A) yielded peptide **2b** (1.4 mg, 2.53 µmol) in 10% yield. ESI-MS (positive mode) = 553.1061 $[M+H]^+$ (calcd. m/z: 553.1057).

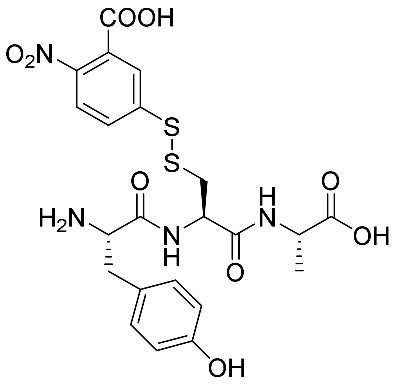

2b

Synthesis of peptide 2c

Procedure: Cysteine peptide **1c** (4.3 mg, 12.1 µmol) was dissolved in DMF (0.175 mL) along with Et_3N (33.7 µL, 242 µmol, 20 eq). To the stirred solution was added a solution of Ellman´s reagent (9.6 mg, 24.2 µmol, 2 eq) in DMF (0.175 mL). Final peptide concentration was 34.6 mM. The reaction mixture was incubated at room temperature for 15 min. Purification *via* semi-preparative HPLC (method A) yielded peptide **2c** (1.1 mg, 2.0 µmol) in 16% yield. ESI-MS (positive mode) = 553.1061 $[M+H]^+$ (calcd. m/z: 553.1057).

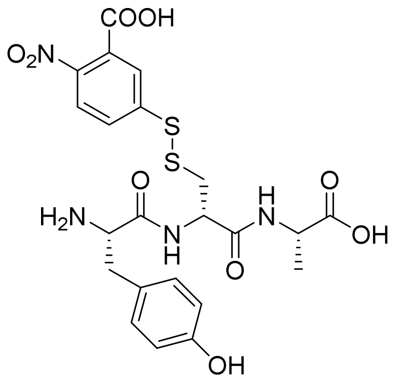

2c

Synthesis of peptide 2d

Procedure: Cysteine peptide **1d** as white trifluoroacetate (18 mg, 10.6 µmol) was dissolved in DMF (0.4 mL) along with Et_3N (29.6 µL, 212 µmol, 20 eq). To the stirred solution was added a solution of Ellman´s reagent (8.7 mg, 21.2 µmol, 2 eq) in DMF (0.4 mL). Final peptide concentration was 13.3 mM. The reaction mixture was incubated at room temperature for 15 min. Purification *via* semi-preparative HPLC (method A) yielded peptide **2d** (12.2 mg, 6.4 µmol) in 61% yield. ESI-MS (positive mode) = 834.8374 $[M+2H]^{2+}$ (calcd. m/z: 834.8427).

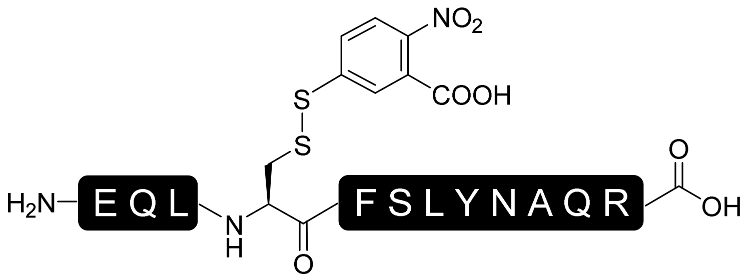

2d

Synthesis of peptide 2e

Procedure: Cysteine peptide **1e** as white trifluoroacetate (8 mg, 5.9 µmol) was dissolved in DMF (225 mL) along with Et_3N (17 µL, 118 µmol, 20 eq). To the stirred solution was added a solution of Ellman´s reagent (4.7 mg, 11.8 µmol, 2 eq) in DMF (225 mL). Final peptide concentration was 13.1 mM. The reaction mixture was incubated at room temperature for 15 min. Purification *via* semi-preparative HPLC (method A) yielded peptide **2e** (2.6 mg, 1.7 µmol) in 28 % yield. ESI-MS (positive mode) = 780.3268 $[M+2H]^{2+}$ (calcd. m/z: 780.3269).

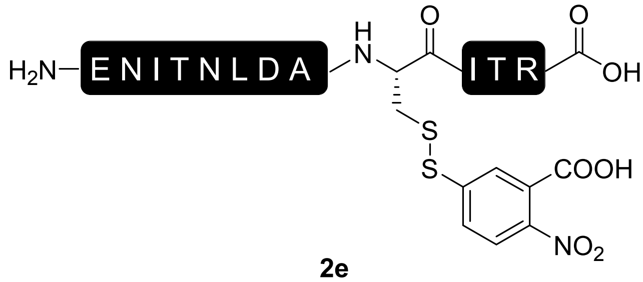

2e

Synthesis of peptide 4b (*O*,*O*-bis(2-cyanoethyl) *S*-cysteine phosphorothiolate peptide)

Procedure in DMF: Ellman´s labelled cysteine peptide **2a** (1.9 mg, 1.5 µmol) was dissolved in DMF (150 µL) and a solution of phosphite **3b** (1.8 mg, 7.5 µmol, 5 eq) in DMF (150 µL) was added. Final peptide concentration was 5 mM. The reaction mixture was incubated at room temperature for 16 h. Purification *via* semi-preparative HPLC (method A) yielded peptide **4b** (0.7 mg, 0.55 µmol) in 37% yield. ESI-MS (positive mode) = 469.7230 $[M+2H]^{2+}$ (calcd. m/z: 469.7251). ^{31}P-NMR (243 MHz, D_2O at pH 7.5) δ = 29.88 ppm.

Procedure in 50 mM Tris Buffer pH 7.2 / MeCN (2:3): Ellman´s labelled cysteine peptide **2a** (1.9 mg, 1.5 µmol) was dissolved in 50 mM Tris Buffer at pH 7.2 (150 µL) and a solution of phosphite **3b** (1.8 mg, 7.5 µmol, 5 eq) in MeCN (150 µL) was added. Final peptide concentration was 5 mM. The reaction mixture was incubated at room temperature for 3 h. Purification *via* semi-preparative HPLC (method A) yielded peptide **4b** (0.8 mg, 0.62 µmol) in 43% yield.

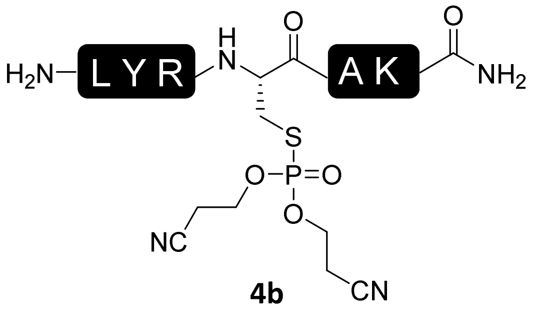

4b

Synthesis of peptide 4d (*O*,*O*-bis(1-(2-nitrophenyl)ethyl)*S*-cysteine phosphorothiolate)

Procedure in DMF: Ellman´s labelled cysteine peptide **2a** (1 mg, 0.8 µmol) was dissolved in DMF (200 µL) and a solution of phosphite **3d** (2.0 mg, 3.9 µmol, 5 eq) in DMF (200 µL) was added. Final peptide concentration was 1.9 mM. The reaction mixture was incubated at room temperature for 16 h. Purification *via* semi-preparative HPLC (method A) yielded peptide **4d** (0.6 mg, 0.4 µmol) in 55% yield. ESI-MS (positive mode) = 565.7439 $[M+2H]^{2+}$ (calcd. m/z: 565.7463. ^{31}P-NMR (243 MHz, D_2O at pH 7.5) δ = 28.40, 27.55, 27.50, 26.79 ppm.

Procedure in 50 mM Tris Buffer pH 7.2 / MeCN (2:3): Ellman´s labelled cysteine peptide **2a** (1 mg, 0.8 µmol) was dissolved in 50 mM Tris Buffer at pH 7.2 (175 µL) and a solution of phosphite **3d** (2.0 mg, 3.9 µmol, 5 eq) in MeCN (225 µL) was added. Final peptide concentration was 1.9 mM. The reaction mixture was incubated at room temperature for 3 h. Purification *via* semi-preparative HPLC (method A) yielded peptide **4d** (0.3 mg, 0.2 µmol) in 27% yield.

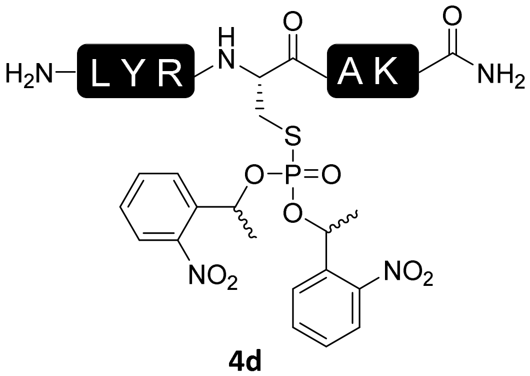

4d

Synthesis of peptide 4e (*O*,*O*-bis((4-((2,5,8,11,14,17-hexaoxanonadecan-19-yl)oxy)-5-methoxy-2-nitrobenzyl) *S*-cysteine phosphorothiolate)

Procedure in DMF: Ellman´s labelled cysteine peptide **2a** (1.5 mg, 1.2 µmol) was dissolved in DMF (300 µL) and a solution of phosphite **3e** (8.7 mg, 6.0 µmol, 5 eq) in DMF (300 µL) was added. Final peptide concentration was 2.0 mM. The reaction mixture was incubated at room temperature for 16 h. Purification *via* semi-preparative HPLC (method A) yielded peptide **4e** (0.65 mg, 0.31 µmol) in 27% yield. ESI-MS (positive mode) = 875.9098 $[M+2H]^{2+}$ (calcd. m/z: 875.9091). ^{31}P-NMR (243 MHz, D_2O at pH 7.5) δ = 30.34 ppm.

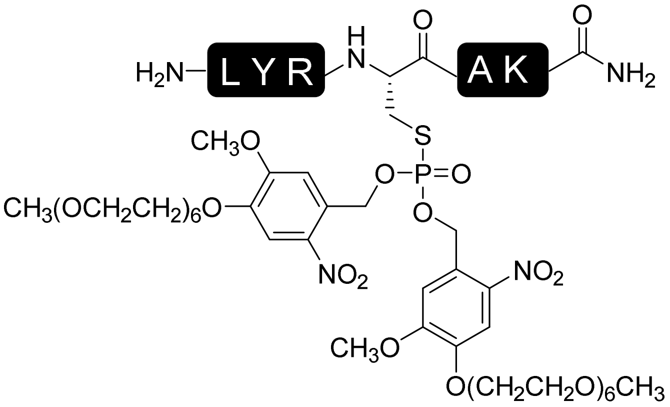

4e

Synthesis of peptide 5e (*O*-(4-((2,5,8,11,14,17-hexaoxanonadecan-19-yl)oxy)-5-methoxy-2-nitrobenzyl) *S*-cysteine phosphorothiolate)

Procedure in DMF: Ellman´s labelled cysteine peptide **2a** (1.5 mg, 1.2 μmol) was dissolved in DMF (300 μL) and a solution of phosphite **3e** (8.7 mg, 6.0 μmol, 5 eq) in DMF (300 μL) was added. Final peptide concentration was 2.0 mM. The reaction mixture was incubated at room temperature for 16 h. Purification *via* semi-preparative HPLC (method A) yielded peptide **5e** (0.64 mg, 0.39 μmol) in 34% yield. ESI-MS (positive mode) = 646.3013 $[M+2H]^{2+}$ (calcd. m/z: 646.3038). ^{31}P-NMR (243 MHz, D_2O at pH 7.5) δ = 18.81 ppm.

*Procedure in_In 50 mM Tris Buffer pH 8.0:*_Ellman´s labelled cysteine peptide **2a** (2 mg, 1.5 μmol) was dissolved in 50 mM Tris Buffer at pH 8.0 (400 μL) and a solution of phosphite **3e** (10.9 mg, 7.5 μmol, 5 eq) in the same buffer (400 μL) was added. Final peptide concentration was 1.9 mM. The reaction mixture was incubated at room temperature for 3 h. Purification *via* semi-preparative HPLC (method A) yielded peptide **5e** (0.92 mg, 0.56 μmol) in 38% yield.

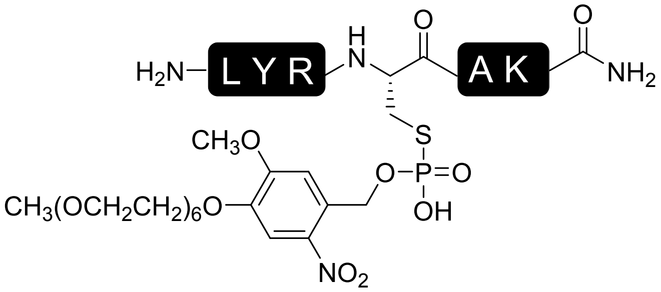

5e

Synthesis of peptide 4l

Procedure: Ellman´s labelled cysteine peptide **2d** (1.7 mg, 0.9 µmol) was dissolved in DMF (225 µL) and a solution of phosphite **3d** (2.4 mg, 4.5 µmol, 5 eq) in DMF (225 µL) was added. Final peptide concentration was 1.8 mM. The reaction mixture was incubated at room temperature for 16 h. Purification *via* semi-preparative HPLC (method A) yielded peptide **4l** (1.0 mg, 0.48 µmol) in 53% yield. ESI-MS (positive mode) = 925.3800 $[M+2H]^{2+}$ (calcd. m/z: 925.3844).

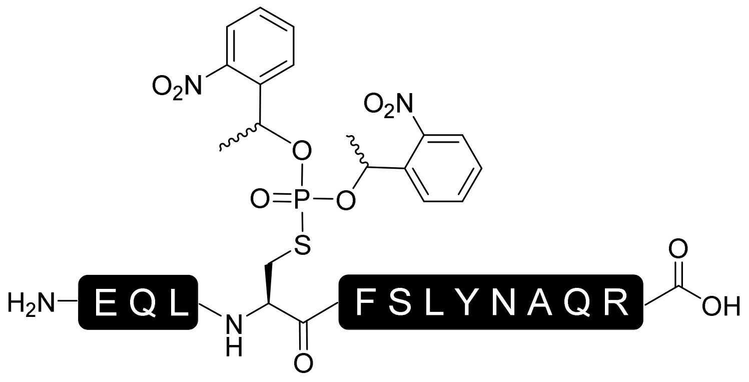

4l

Synthesis of peptide 4m

Procedure: Ellman´s labelled cysteine peptide **2e** (1.3 mg, 0.8 µmol) was dissolved in DMF (200 µL) and a solution of phosphite **3d** (2.2 mg, 4.2 µmol, 5 eq) in DMF (200 µL) was added. Final peptide concentration was 2.1 mM. The reaction mixture was incubated at room temperature for 16 h. Purification *via* semi-preparative HPLC (method A) yielded peptide **4m** (0.67 mg, 0.39 µmol) in 47 % yield. ESI-MS (positive mode) = 870.8687 $[M+2H]^{2+}$ (calcd. m/z: 870.8686).

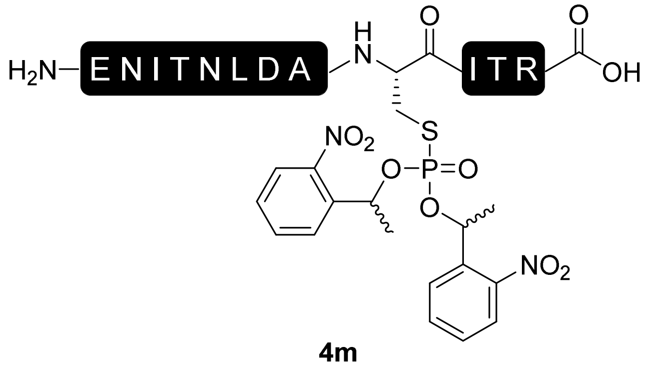

4m

Synthesis of peptide 6a

Procedure: Phosphorothiolate peptide **4d** (1.3 mg, 0.88 µmol) was dissolved in Tris-HCl buffer solution (500 µL). Final peptide concentration was 1.8 mM. The sample was irradiated at 295 nm with a UV lamp for 5 min. Purification *via* semi-preparative HPLC (method B) yielded peptide **6a** as an ammonium acetate salt (0.6 mg, 0.57 µmol) with a purity criteria ≥ 90% based on UPLC-UV and in 65% yield. ESI-MS (positive mode) = 416.6968 $[M+2H]^{2+}$ (calcd. m/z: 416.6986). ^{31}P-NMR (243 MHz, D_2O at pH 7.5) δ = 12.02 ppm.

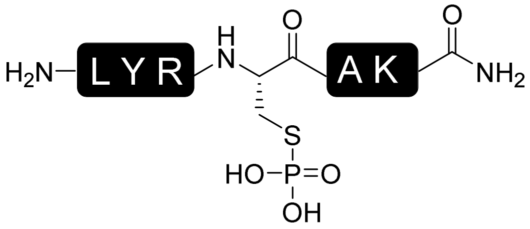

6a

Synthesis of peptide 6b

Procedure: Phosphorothiolate peptide **4l** (1.0 mg, 0.48 µmol) was dissolved in Tris-HCl buffer solution (500 µL). Final peptide concentration was 0.96 mM. The sample was irradiated at 295 nm with a UV lamp for 5 min. Purification *via* semi-preparative HPLC (method B) yielded peptide **6b** as an ammonium acetate salt (0.6 mg, 0.34 µmol) with a purity criteria ≥ 74% based on UPLC-UV and in 73% yield. ESI-MS (positive mode) = 776.3347 $[M+2H]^{2+}$ (calcd. m/z: 776.3367).

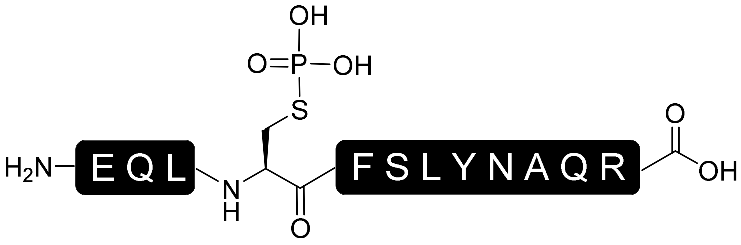

6b

Synthesis of peptide 6c

Procedure: Phosphorothiolate peptide **4m** (0.66 mg, 0.38 μmol) was dissolved in Tris-HCl buffer solution (400 μL). Final peptide concentration was 0.95 mM. The sample was irradiated at 295 nm with a UV lamp for 5 min. Peptide **6c** was analyzed by HPLC-UV and –MS without further purification. ESI-MS (positive mode) = 721.8212 $[M+2H]^{2+}$ (calcd. m/z: 721.8209).

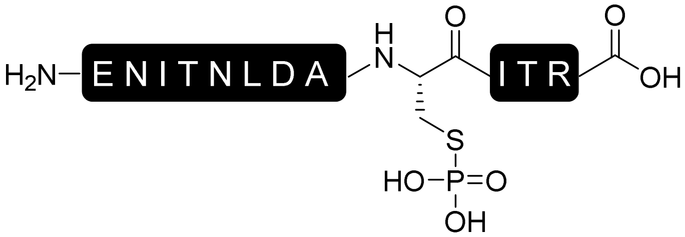

6c
